# Effect of B_4_C Content and Heat Treatment on the Microstructure, Mechanical Properties, and Tribological Behavior of Alumix–B_4_C Composites

**DOI:** 10.3390/ma19173786

**Published:** 2026-09-06

**Authors:** Marcin Madej, Beata Leszczyńska-Madej, Anna Puzia, Anna Wąsik, Marcin Goły

**Affiliations:** 1 Faculty of Metals Engineering and Industrial Computer Science, AGH University of Krakow, 30 Mickiewicza Ave, 30-059 Krakow, Poland; marcing@agh.edu.pl; 2 Faculty of Non-Ferrous Metals, AGH University of Krakow, 30 Mickiewicza Ave, 30-059 Krakow, Poland; puziaania@student.agh.edu.pl (A.P.); anna.wasik@agh.edu.pl (A.W.)

**Keywords:** Alumix 431, B_4_C reinforcement, aluminum matrix composites, precipitation hardening, tribological behavior, wear mechanisms

## Abstract

The present study investigates the combined effect of B_4_C content and heat treatment on the microstructure, mechanical properties, and tribological behaviour of Alumix 431 matrix composites consolidated by field-assisted sintering technology/spark plasma sintering (FAST/SPS). Composites containing 5 wt.% and 10 wt.% B_4_C were examined in the as-sintered, solution-treated, and artificially aged conditions. Microstructural evolution was characterized by SEM/EDS, XRD, and TEM, while hardness, compression, and dry-sliding tests were used to evaluate material performance. Artificial ageing promoted the formation of fine MgZn_2_ and (Cu,Zn)Mg precipitates and substantially strengthened the matrix. The highest mechanical performance was obtained for the aged 5 wt.% B_4_C composite, reaching 172 HV1 and a maximum compressive stress of 742 ± 12 MPa. Increasing the B_4_C content to 10 wt.% did not further improve the mechanical properties because of greater microstructural heterogeneity. In contrast, the higher B_4_C content became beneficial under tribological loading after heat treatment, with the aged 10 wt.% B_4_C composite exhibiting the lowest wear rate after 500 m. Worn-surface analysis revealed predominantly abrasive wear accompanied by tribo-oxidation. The results demonstrate that composite performance is governed by the interaction between B_4_C content and the precipitation-strengthened matrix rather than by reinforcement content alone.

## 1. Introduction

Aluminum and aluminum-alloy matrix composites reinforced with ceramic particles, including B_4_C, remain one of the most actively investigated groups of lightweight materials. This interest stems from their potential to improve mechanical and tribological properties and tailor the microstructure while maintaining a low material density. In particular, boron carbide-reinforced composites have attracted considerable interest for demanding applications in the aerospace, automotive, and defense industries, as well as in nuclear engineering [1,2,3]. The available literature indicates that increasing the B_4_C content may improve the hardness and wear resistance of aluminum matrix composites, provided that the particles are uniformly distributed and the interfaces are of sufficient quality [3,4,5,6]. This relationship has also been confirmed in recent studies on Al2011/B_4_C and Al2024/B_4_C composites, which reported changes in hardness, mechanical properties, and wear resistance with increasing ceramic-phase content [7,8]. In powder metallurgy-processed Al–B_4_C composites, increasing the B_4_C content to 30 wt.% resulted in an increase in hardness from approximately 28 HV to 75 HV, corresponding to a 167% improvement, and a 76.7% reduction in the specific wear rate under a load of 7 N, reaching 0.00042 mm^3^/N·m [4]. For AA5083 matrix composites, increasing the B_4_C content from 5 to 10 wt.% was associated with an approximately 40% reduction in the wear rate. The presence of B_4_C particles also influences the morphology of the worn surface and the formation of a mechanically mixed layer containing oxidation products and material transferred from the counterbody. This layer may reduce direct contact between the sliding surfaces and stabilize the wear process [3]. However, the effect of increasing the B_4_C content is not unequivocally beneficial, as particle agglomeration, increased porosity, or instability of the mechanically mixed layer may impair composite performance [3,5,6]. Therefore, B_4_C should be regarded not only as a conventional reinforcing phase that increases composite hardness, but also as a constituent that actively governs its tribological response by affecting the operative wear mechanisms and the formation and stability of the mechanically mixed layer.

However, the presence of ceramic particles alone does not exhaust the possibilities for tailoring composite properties. The microstructural condition of the matrix is equally important and, in precipitation-hardenable aluminum alloys, can be modified through appropriately selected solution treatment and aging. Such heat treatment affects the nucleation and growth of precipitates and, consequently, their type, size, number density, and spatial distribution within the matrix. In Al–Zn–Mg(–Cu) alloys, precipitation is generally described by the following sequence: supersaturated solid solution → Guinier–Preston (GP) zones → metastable η′ phase → equilibrium η phase (MgZn_2_). However, the detailed precipitation pathway and kinetics depend on the alloy composition as well as the aging temperature and time [9,10,11]. Consequently, composite properties are governed not only by the content and distribution of B_4_C particles but also by the evolution of the precipitation-hardenable matrix microstructure. The incorporation of ceramic particles into precipitation-hardenable aluminum alloys may significantly modify the aging response, with the direction and magnitude of this effect depending on both the matrix composition and the type and content of the reinforcement. In Al–Cu matrix composites, ceramic particles have been reported to promote vacancy trapping at dislocations and interfaces, resulting in a pronounced delay in GP-zone formation without substantially affecting the final volume fraction of precipitates [12,13]. In Al–Mg–Si matrix composites, the influence of the reinforcing phase on the early stages of aging was less pronounced because solute atom–vacancy pairs participated in the formation of the initial clusters. At the same time, the increased dislocation density around the particles could promote the heterogeneous nucleation of subsequently formed, partially coherent precipitates [12]. More complex relationships have been reported directly for B_4_C/6061Al composites. Increasing the B_4_C content from 3 to 12 vol.% accelerated the attainment of peak hardness, which was attributed to an increase in dislocation density caused by the mismatch in the coefficients of thermal expansion between the matrix and the reinforcement. At the same time, the presence of B_4_C suppressed GP-zone formation, altered the precipitation sequence, and reduced the overall amount of precipitates. Increasing the B_4_C content from 3 vol.% to 12 vol.% reduced the enthalpy associated with precipitation reactions by approximately 50%. This effect was attributed, among other factors, to Mg segregation at the matrix–B_4_C interfaces and restricted Mg and Si diffusion resulting from the disruption of matrix continuity by the ceramic particles [14]. The importance of interfacial reactions was also demonstrated for B_4_C/6061Al composites containing up to 30 wt.% B_4_C, in which Mg consumption through reactions occurring at the matrix–reinforcement interfaces reduced the precipitation-hardening capability of the material [15]. The influence of the reinforcing phase on precipitation processes is also highly localized. In B_4_C/7075Al composites, matrix regions directly adjacent to the particles exhibited a high dislocation density, fewer but larger GP zones, and enhanced heterogeneous nucleation of the η′ and η phases compared with regions located farther from the interface [16]. This indicates that B_4_C is not a passive constituent of the composite but may actively govern the kinetics, sequence, and spatial heterogeneity of precipitation processes. Consequently, the effects of heat treatment should be considered in conjunction with the reinforcement content, the condition of the matrix–B_4_C interfaces, and the local matrix microstructure.

The available literature indicates that the effects of B_4_C content, composite microstructure, and heat treatment on material properties have generally been investigated separately. Review articles have shown that the hardness and wear resistance of Al–B_4_C composites depend on the content and distribution of the particles, the quality of the interfaces, the processing parameters, and the tribological test conditions [3,17]. Studies focusing on Al–B_4_C interfaces have further demonstrated that the course of interfacial reactions and the types of phases formed depend on the matrix composition, processing temperature, and the surface condition and size of the B_4_C particles [18]. In addition, Toptan et al. [19] demonstrated that solution treatment followed by aging increased the hardness of an AA6063–10 wt.% B_4_C composite from 48 to 68 HV without causing noticeable changes in the thin TiC and TiB_2_ reaction layers at the matrix–reinforcement interfaces. However, that study did not investigate tribological properties or compare composites containing different amounts of B_4_C. More recent research on a B_4_C/6061Al composite demonstrated that the aging parameters affected not only the formation of the β″ and β′ phases but also hardness, wear mechanisms, and wear severity [20]. These findings confirm the need to consider the evolution of the matrix microstructure together with the mechanical and tribological responses of the composite. Nevertheless, they do not provide a systematic comparison of composites with different B_4_C contents before and after the same heat-treatment cycle.

In the present study, Alumix 431 matrix composites containing 5 wt.% and 10 wt.% B_4_C were comprehensively compared in both the as-sintered and heat-treated conditions, with the latter comprising solution treatment followed by artificial aging. This experimental design enabled the effects of B_4_C content and heat treatment to be distinguished and their combined influence on the microstructure, hardness, compressive behavior, and tribological properties to be evaluated. The novelty of this study lies in establishing systematic relationships among B_4_C content, the microstructural condition of the precipitation-hardenable matrix, and the resulting mechanical and tribological behavior of the composites.

## 2. Materials and Methods

### 2.1. Starting Materials and Composite Fabrication

Commercial Alumix 431 powder without solid lubricant additives (Ecka Granules, Velden, Germany), intended for consolidation by powder metallurgy routes, was used as the matrix material. Its chemical composition is given in Table 1. Alumix 431 belongs to the 7xxx series of aluminum alloys, with zinc, magnesium, and copper as the principal alloying elements. This composition enables the mechanical properties of the matrix to be tailored through solution treatment and aging, which provided the basis for the heat-treatment procedure applied in this study.

**Figure 1:**
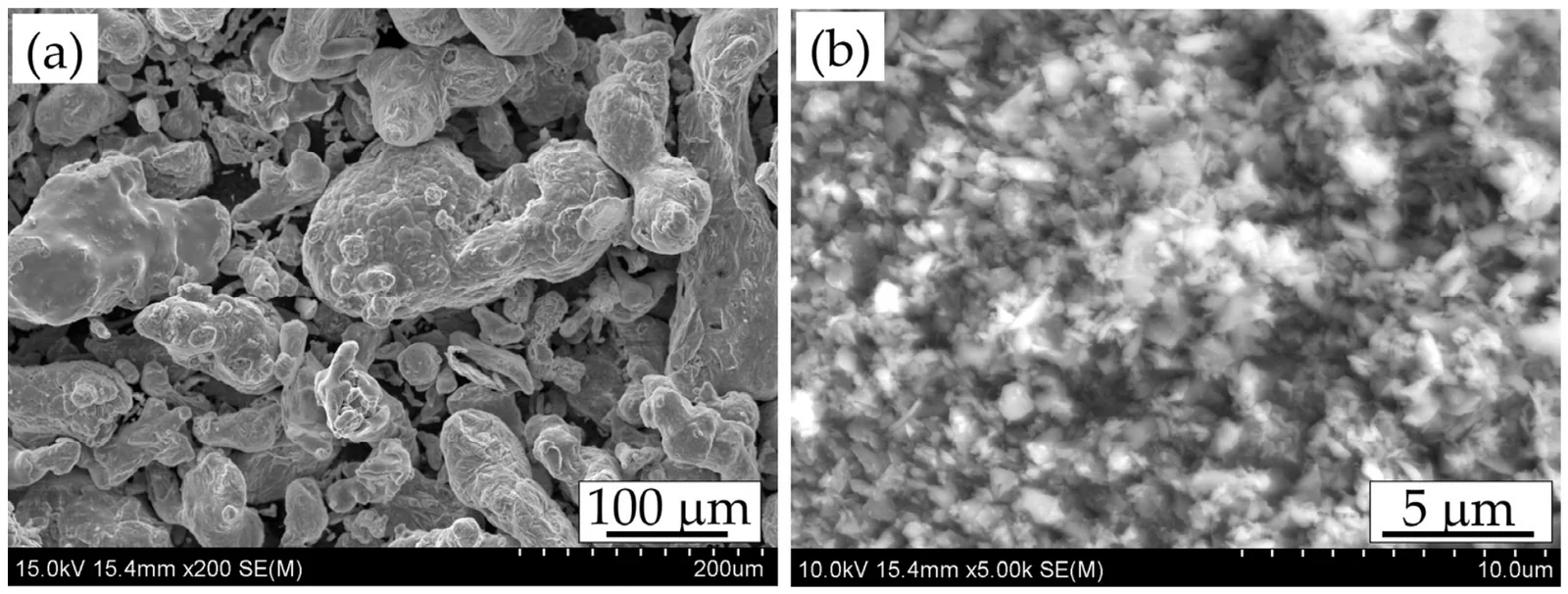
SEM images showing the morphology of the starting powders: (**a**) Alumix 431 and (**b**) B_4_C.

The Alumix 431 powder was produced by air atomization. The particles had a size below 86 µm and exhibited an irregular morphology, with locally rounded features and relatively rough surfaces. Such morphology is characteristic of air-atomized aluminum alloy powders and promotes mechanical interlocking between particles and their compaction during consolidation. Boron carbide powder (B_4_C; KAMB Import-Export, Warsaw, Poland), produced by powder synthesis, was used as the reinforcing phase. The B_4_C particles had an average size below 0.8 µm and an irregular, angular morphology. B_4_C was added to the matrix powder in amounts of 5 wt.% and 10 wt.%, resulting in two composite series differing in ceramic-phase content. The morphologies of the starting powders are shown in Figure 1.

The Alumix 431 and B_4_C powder mixtures were homogenized for 60 min in a Turbula T2F mixer (WAB, Muttenz, Switzerland) and then manually pre-compacted in a graphite die with an inner diameter of 40 mm. Consolidation was performed by FAST/SPS using an HP D 25/3 system (FCT Systeme, Rauenstein, Germany) under a vacuum of approximately 5 × 10^−2^ mbar. The sintering process was carried out at 500 °C under a pressure of 80 MPa, with a heating rate of 100 °C/min and a dwell time of 10 min. The resulting compacts were cylindrical, with a diameter of 40 mm and a height of approximately 10 mm. A detailed description of the composite fabrication procedure and the effects of the FAST/SPS process parameters on densification, microstructure, and selected properties was presented in our previous study [21]. The relative densities of the as-sintered composites were determined hydrostatically according to Archimedes’ principle. Values of 96.49% and 96.85% were obtained for the composites containing 5 wt.% and 10 wt.% B_4_C, respectively [21].

### 2.2. Heat Treatment

The composites containing 5 wt.% and 10 wt.% B_4_C were investigated in three conditions: as-sintered by FAST/SPS, solution-treated, and solution-treated followed by artificial aging. Solution treatment was carried out at 480 °C for 1 h, followed by water quenching. For the full heat-treatment cycle, the solution-treated samples were subsequently artificially aged at 160 °C for 8 h. The solution-treatment temperature was selected based on literature data for sintered Alumix 431 and B_4_C-reinforced Al–Zn–Mg–Cu matrix composites, for which solution treatment is typically conducted within the temperature range of 470–480 °C. The artificial-aging parameters were established based on previous experimental work and reported precipitation behavior at elevated aging temperatures [22,23,24].

The sample designations and the corresponding processing and heat-treatment conditions are summarized in Table 2. For clarity, the sample codes listed in the table are used throughout the remainder of this study.

### 2.3. Microstructural and Phase-Composition Characterization

Samples intended for microstructural examination were ground using abrasive papers of progressively decreasing grit size and subsequently polished with diamond suspensions until surfaces free of scratches and other preparation-induced artifacts were obtained. To reveal the microstructure, the metallographic specimens were etched using Keller’s reagent composed of 190 mL H_2_O, 5 mL HNO_3_, 3 mL HCl, and 2 mL HF. Preliminary microstructural examination, including the assessment of B_4_C particle distribution, particle agglomeration, porosity, and other discontinuities, was performed using an OLYMPUS GX51 optical microscope (OLYMPUS GX51, Tokyo, Japan). Detailed microstructural characterization was conducted using a Hitachi SU-70 scanning electron microscope (Hitachi, Tokyo, Japan) equipped with a NORAN System 7 energy-dispersive X-ray spectroscopy (EDS) system (Thermo Fisher Scientific, Waltham, MA, USA). The observations were performed at an accelerating voltage of 15 kV using secondary-electron (SE) and backscattered-electron (BSE) detectors. EDS analysis was used to determine the local chemical composition of selected microstructural regions, with particular emphasis on the matrix–B_4_C interfacial regions. Transmission electron microscopy investigations were carried out using a JEOL JEM-ARM200F NEOARM transmission electron microscope (JEOL Ltd., Tokyo, Japan) operated at an accelerating voltage of 200 kV. Thin foils were prepared using a PIPS II Model 695 precision ion polishing system (Gatan, Inc., Pleasanton, CA, USA). The phase composition was analysed by X-ray diffraction (XRD) using a Bruker D8 Advance diffractometer (Bruker, Karlsruhe, Germany) equipped with a cobalt X-ray source. The measurements were performed using Co Kα radiation with a wavelength of λ = 1.79 Å over a 2θ range of 30–120°, with a step size of 0.02°. Both TEM and XRD investigations were performed for the composite containing 5 wt.% B_4_C in the as-sintered FAST/SPS condition, after solution treatment, and after solution treatment followed by artificial aging.

### 2.4. Hardness Measurements

The hardness of the composites was measured using the Vickers method in accordance with PN-EN ISO 6507-1 (ISO 6507-1:2018) [25] using an INNOVATEST hardness tester (INNOVATEST Europe BV, Maastricht, The Netherlands). Measurements were performed on appropriately prepared metallographic specimens under a load of 1 kgf (HV1). For each material variant, ten indentations were made along the sample axis to account for local microstructural variations associated with the presence of the ceramic particles. The results are reported as mean values with standard deviations.

### 2.5. Uniaxial Compression Testing

The mechanical behavior of the composites under uniaxial compression was investigated using a Zwick/Roell universal testing machine (ZwickRoell GmbH & Co. KG, Ulm, Germany). The tests were performed in accordance with ASTM E9 [26] using cylindrical specimens with a height-to-diameter ratio of approximately 1.5. The end faces of the specimens were ground and polished to ensure their parallelism and uniform load transfer. Three specimens were tested for each material variant. During testing, stress–strain curves were recorded. Based on these curves, the compressive stress at 10% strain and the maximum compressive stress were determined. The results are reported as mean values with standard deviations.

### 2.6. Tribological Testing

Tribological tests were conducted under dry sliding conditions using a T-05 block-on-ring tribometer (ITEE, Radom, Poland). Composite specimens in the form of stationary blocks with dimensions of 4 mm × 4 mm × 20 mm were pressed against a rotating 100Cr6 steel ring counterbody with a hardness of 53 ± 2 HRC. The tests were performed under a normal load of 50 N and at a rotational speed of 136 rpm. For each material variant, two sliding distances, 250 and 500 m, were applied. Three specimens were tested for each material variant and for each sliding distance (n = 3). The friction force was continuously recorded and used to calculate the coefficient of friction. Wear resistance was assessed based on the loss of mass of the specimens. After testing, the worn surfaces were examined using a VHX-4000 digital microscope (KEYENCE Corporation, Osaka, Japan) to identify the dominant wear mechanisms.

## 3. Results

### 3.1. Microstructure and Phase Composition

**Figure 2:**
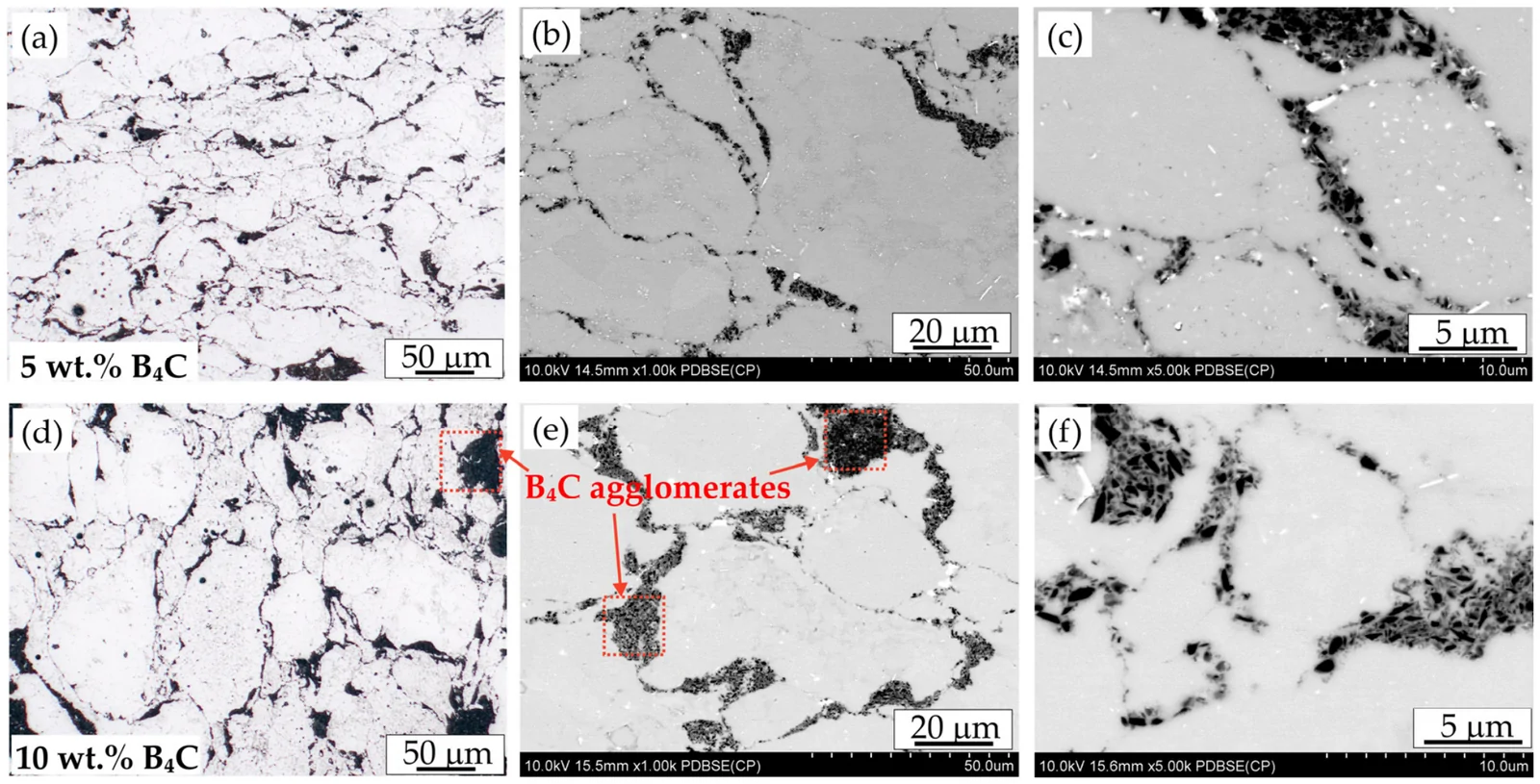
Microstructure of the Alumix 431 composites containing 5 wt.% B_4_C (A1; **a**–**c**) and 10 wt.% B_4_C (A2; **d**–**f**) in the as-sintered condition: (**a**,**d**) LM images; (**b**,**c**,**e**,**f**) SEM-BSE images at progressively higher magnifications.

Figure 2 presents representative microstructures of Alumix 431 composites containing 5 wt.% and 10 wt.% B_4_C in the as-sintered FAST/SPS condition. In both materials, the aluminium alloy matrix is continuous, whereas the B_4_C particles are located predominantly in interparticle regions, forming band-like arrangements that reflect the boundaries of the original Alumix 431 powder particles. This distribution of the ceramic phase results from powder-mixture preparation and subsequent consolidation: the substantially finer B_4_C particles become located on the surfaces of the larger matrix powder particles and, after sintering, remain concentrated along their original boundaries. A similar distribution of B_4_C was reported previously for AA7075–B_4_C composites fabricated by FAST/SPS [21,24]. In the composite containing 5 wt.% B_4_C, the reinforcing particles form relatively thin and predominantly discontinuous bands, although local agglomerates are also present. Increasing the B_4_C content to 10 wt.% leads to a clear increase in the fraction of ceramic-rich regions and to the formation of larger and more compact agglomerates (Figure 2d–f). Consequently, the distribution of the reinforcing phase becomes more heterogeneous. The accumulation of ceramic particles in interparticle regions may locally reduce contact between matrix particles during sintering, thereby affecting densification and the formation of metallurgical bonds.

**Figure 3:**
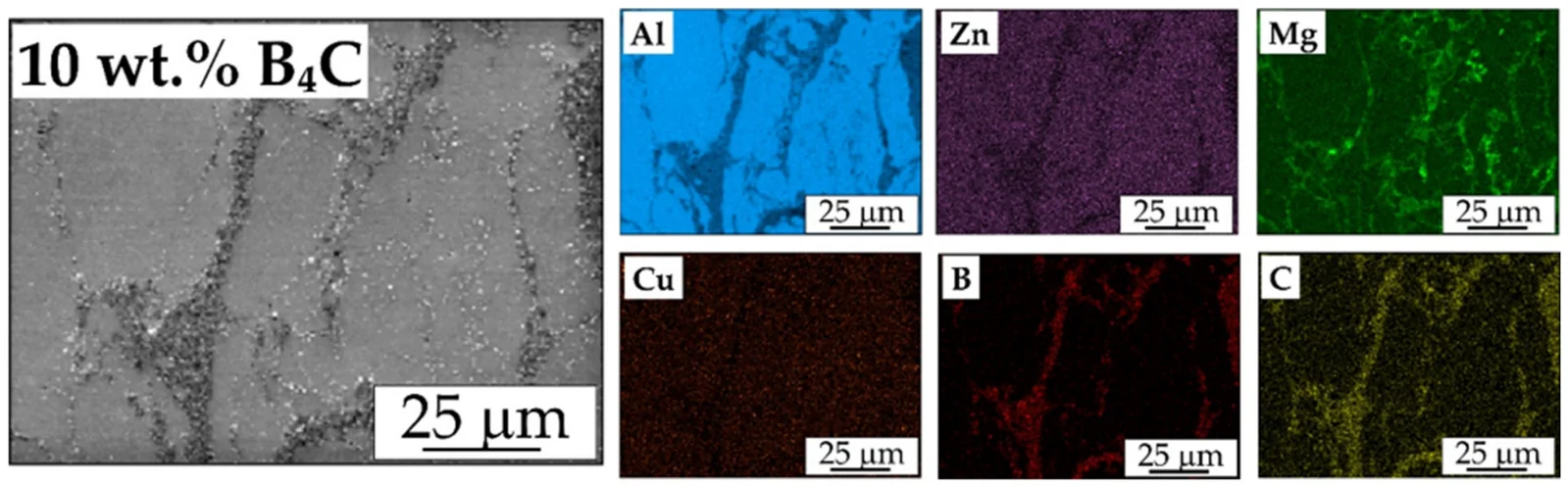
SEM-BSE image and corresponding EDS elemental maps of Al, Zn, Mg, Cu, B, and C for the Alumix 431–10 wt.% B_4_C composite in the as-sintered condition (A2).

The identification of the dark regions as B_4_C agglomerates is supported by SEM/EDS elemental distribution maps obtained for the Alumix 431–10 wt.% B_4_C composite (Figure 3). These regions show pronounced boron enrichment accompanied by a corresponding decrease in the aluminium signal intensity. The carbon signal is less distinct because of the limited quantitative accuracy of EDS analysis for light elements; nevertheless, its spatial distribution remains consistent with the locations of boron-rich regions. Local Mg enrichment was also observed near B_4_C agglomerates. Based on the SEM/EDS maps alone, it cannot be determined whether this enrichment results from Mg segregation, the presence of Mg-rich oxygen-containing regions, or possible interfacial reaction products. Fine phase constituents appearing bright in the BSE images are also present within the matrix. The local heterogeneity in the Cu and Zn distributions observed in the SEM/EDS maps is characteristic of the multicomponent Alumix 431 powder and is consistent with previous studies of this system [24]. However, the individual phases cannot be unambiguously identified based solely on BSE contrast and SEM/EDS chemical analysis. Their phase composition is therefore discussed further in light of the XRD and TEM results.

**Figure 4:**
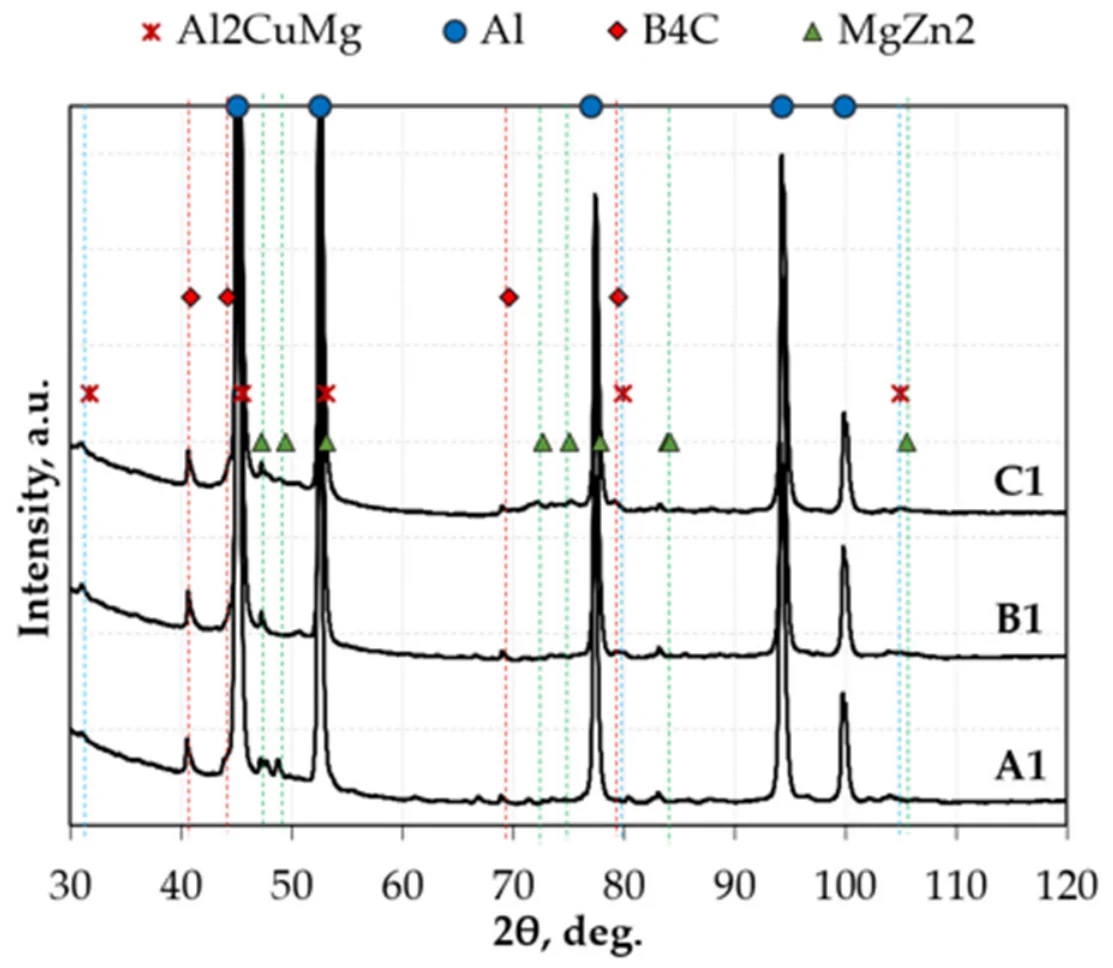
X-ray diffraction patterns of the Alumix 431–5 wt.% B_4_C composite in the as-sintered (A1), solution-treated (B1), and solution-treated and artificially aged (C1) conditions.

Figure 4 presents the X-ray diffraction patterns of the Alumix 431–5 wt.% B_4_C composite in the as-sintered FAST/SPS condition (A1), after solution treatment (B1), and after solution treatment followed by artificial aging (C1). In all analysed conditions, reflections originating from the α-Al matrix and the B_4_C reinforcing phase predominate. Weak reflections assigned to the Al_2_CuMg and MgZn_2_ phases were also identified; their low intensity indicates a small contribution of these phases to the composite microstructure. Solution treatment did not result in the appearance of new phases or any substantial change in the diffraction patterns. The persistence of weak intermetallic-phase reflections after solution treatment indicates that their complete dissolution in the matrix cannot be confirmed under the applied conditions. This may result from the heterogeneous distribution of alloying elements in the powder-metallurgy material and from the local limitation of diffusion by B_4_C particles and interfacial regions. After artificial aging, the reflections assigned to the MgZn_2_ phase at 2θ = 72.62°, 75.03°, and 105.57° exhibit slightly higher intensity and are more clearly developed than those in the as-sintered and solution-treated conditions. These changes indicate the development of MgZn_2_ precipitates during aging; however, owing to the low intensity of the reflections, they do not provide a basis for a quantitative assessment of the fraction of this phase.

The observed changes are consistent with the typical heat-treatment response of Al–Zn–Mg–Cu alloys, in which solution treatment promotes the dissolution of coarse secondary phases, whereas subsequent aging leads to the precipitation of Mg- and Zn-rich phases. Chen et al. [22] showed that MgZn_2_ was the predominant secondary phase in the B_4_C/7A04Al composite before solution treatment, and that its dissolution depended on the solution-treatment temperature. The authors also pointed out that ceramic particles may affect the diffusion of alloying elements and the microstructural stability of the composite. The presence of B_4_C may additionally result in locally differentiated precipitation behavior. Wu et al. [16] demonstrated that the immediate vicinity of B_4_C particles exhibited an increased dislocation density, arising, among other factors, from the mismatch in the coefficients of thermal expansion between the matrix and the ceramic phase. Dislocations and Al/B_4_C interfaces may act as sites for the heterogeneous nucleation of η′ precipitates. At the same time, regions close to B_4_C particles were reported to contain fewer but larger GP zones, together with local depletion of Mg and Zn in the matrix due to precipitate growth. The applied aging temperature of 160 °C promotes accelerated precipitation processes. Chen et al. [23] demonstrated that aging at this temperature may accelerate the nucleation of η′ precipitates. At the same time, the aging kinetics of the B_4_C/7A04Al composite were slower than those of the unreinforced alloy, which was attributed to the local consumption of Mg in reactions involving boron-containing impurities associated with B_4_C. Nevertheless, the presence of the ceramic phase did not alter the fundamental precipitation sequence.

**Figure 5:**
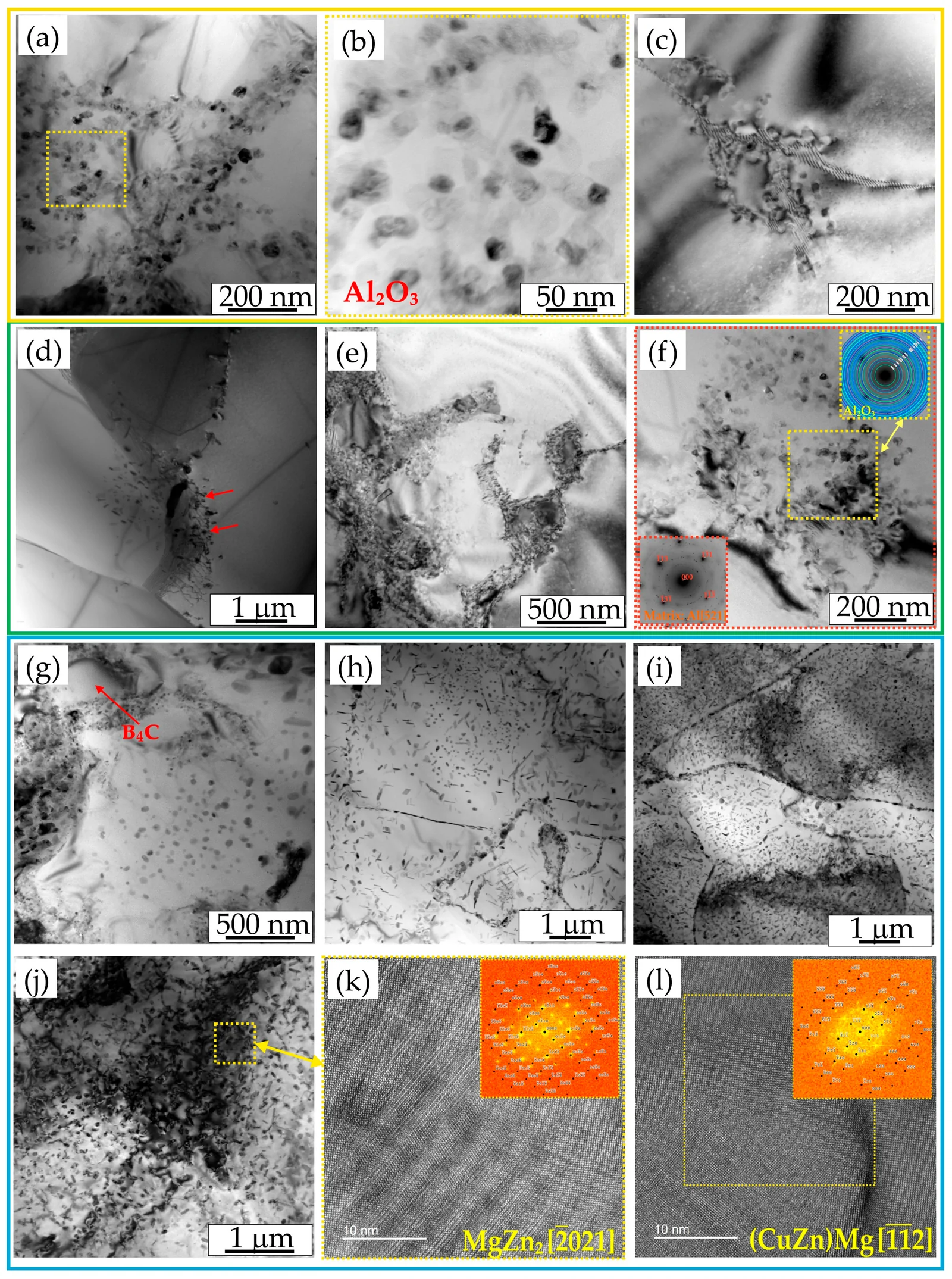
TEM characterization of the Alumix 431–5 wt.% B_4_C composite in different material conditions: (**a**–**c**) as-sintered, (**d**–**f**) solution-treated, and (**g**–**l**) solution-treated and artificially aged. Panels (**b**,**f**) show the identification of Al_2_O_3_ particles, while (**g**) shows a B_4_C particle embedded in the Al matrix. Panels (**h**–**j**) illustrate the distribution and morphology of nanoscale precipitates formed during artificial aging. HRTEM images and corresponding indexed FFT patterns confirm the presence of (**k**) MgZn_2_ and (**l**) (Cu,Zn)Mg precipitates. The arrows and dashed boxes indicate selected particles and regions subjected to detailed analysis.

Owing to the low fraction of secondary phases and the nanoscale size of the precipitates, their detailed characterisation was performed using transmission electron microscopy, electron diffraction, and HRTEM/FFT analysis (Figure 5). In the as-sintered composite, numerous nanoscale Al_2_O_3_ particles were observed, occurring individually or in small clusters within the aluminium matrix (Figure 5a–c). Their presence can be associated with the naturally formed oxide layer on the surfaces of aluminium powder particles, which develops during powder production and exposure to the atmosphere. This layer is typically several nanometres thick and may be amorphous or partially crystalline [27,28,29]. During powder consolidation, its continuity is disrupted as a result of plastic deformation of the matrix particles, applied pressure, and thermal stresses arising from the difference in the coefficients of thermal expansion of Al and Al_2_O_3_. In FAST/SPS processing, the fragmentation of the oxide layer may additionally be promoted by local thermal and electrical effects occurring at particle-contact regions [27]. Consequently, the initially continuous surface layer becomes fragmented and remains in the consolidated material in the form of nanoscale Al_2_O_3_ particles. A similar transformation of the native oxide layer into a dispersion of fine oxide particles has been reported in aluminium materials produced by powder-metallurgy routes [27,28]. These particles may provide an additional strengthening contribution by hindering dislocation motion and grain-boundary migration. However, based on the present observations, their contribution to strengthening cannot be quantitatively separated from the effects of B_4_C and the precipitates formed during aging. After solution treatment, nanoscale Al_2_O_3_ particles were still observed, confirming their stability under the applied heat-treatment conditions (Figure 5d–f). In some areas, dislocation lines were visible bending around or terminating at fine particles (Figure 5d). This observation indicates dislocation–particle interactions and their local pinning. In the analysed regions of the as-sintered and solution-treated material, no distinct population of fine precipitates characteristic of precipitation strengthening in Al–Zn–Mg–Cu alloys was observed. This is consistent with the purpose of solution treatment, which is to dissolve the largest possible fraction of Zn-, Mg-, and Cu-rich phases and to obtain a supersaturated solid solution. A pronounced microstructural change was observed after artificial aging. The matrix contained a numerous population of nanoscale precipitates with elongated or dot-like morphologies, depending on their orientation relative to the electron beam (Figure 5h–j). Their presence indicates the development of precipitation processes from the supersaturated solid solution during aging at 160 °C. HRTEM analysis and FFT indexing enabled the identification of two types of precipitates: MgZn_2_ and (Cu,Zn)Mg (Figure 5k,l), while the presence of MgZn_2_ was additionally confirmed by electron diffraction. These results are consistent with the XRD analysis, which showed a slight increase in the intensity of reflections assigned to the MgZn_2_ phase at 2θ = 72.62°, 75.03°, and 105.57° after aging. However, the TEM observations indicate that the transformations occurring during aging involve primarily nanoscale precipitates, whose low volume fraction hinders their unambiguous characterisation by XRD. The two methods therefore provide complementary information: XRD indicates the presence of MgZn_2_ in the bulk material, whereas TEM/HRTEM enables the determination of the morphology, distribution, and phase diversity of the precipitates. The observed transformations are consistent with the precipitation sequence in Al–Zn–Mg–Cu alloys, involving the formation of GP zones, metastable η′ precipitates, and the equilibrium η-MgZn_2_ phase [9]. In B_4_C-containing composites, this process may be locally modified by dislocations generated because of the mismatch in the coefficients of thermal expansion between the matrix and the ceramic phase, as well as by Al/B_4_C interfaces. These defects may act as sites for heterogeneous nucleation of precipitates and affect the aging kinetics of the composite [22,23]. At the same time, Mg segregation or binding at the surfaces of ceramic particles may reduce its availability in the matrix and lead to spatial variations in the precipitation process [22,28,29].

**Figure 6:**
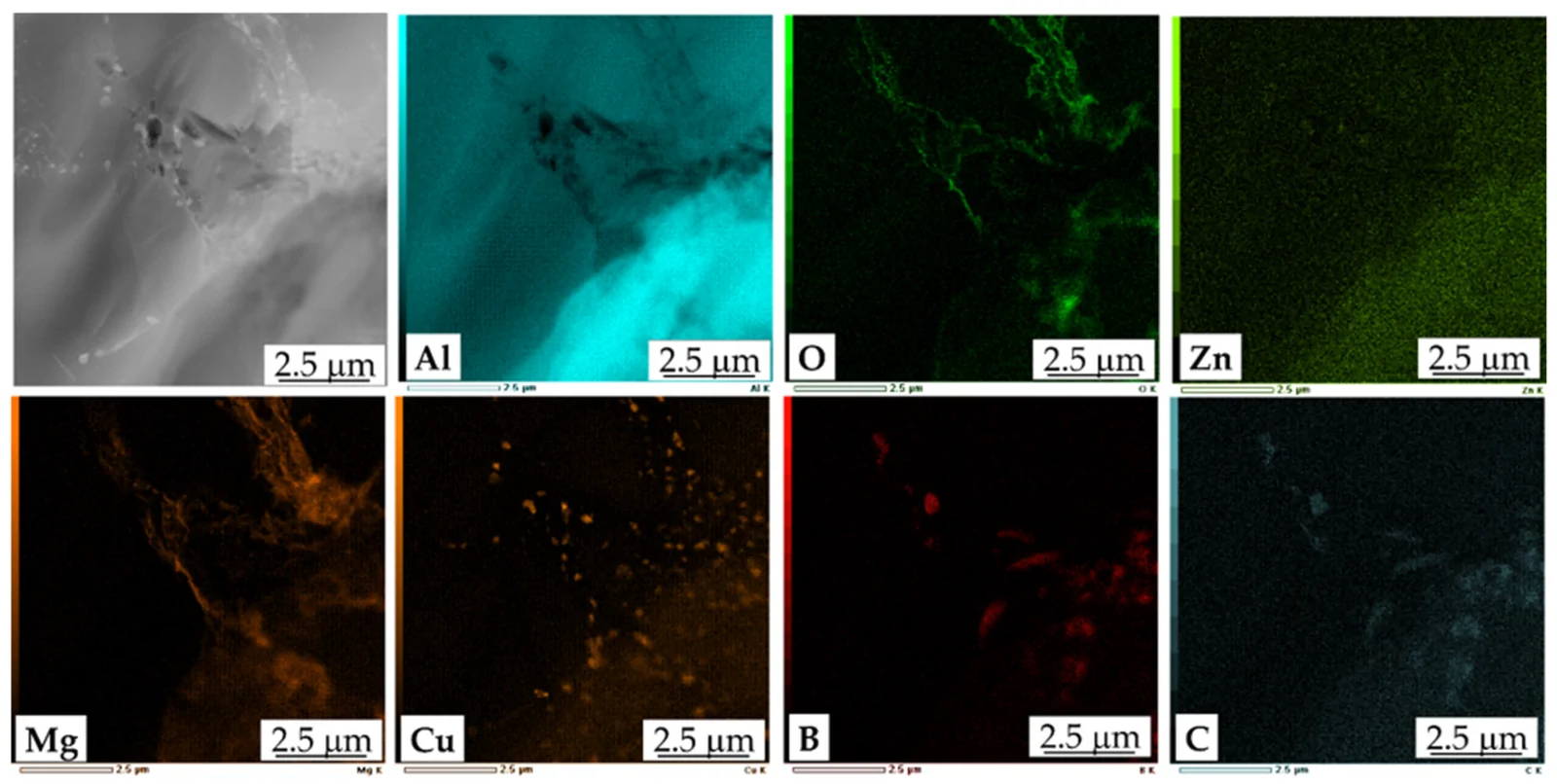
STEM image and corresponding EDS elemental maps of Al, O, Zn, Mg, Cu, B, and C obtained from the Alumix 431–5 wt.% B_4_C composite, confirming the presence of a B_4_C particle embedded in the Al matrix.

The presence of B_4_C in the aluminium matrix was further confirmed by STEM imaging and EDS mapping (Figure 6). The particle area exhibited simultaneous enrichment in B and C, together with a pronounced decrease in the Al signal intensity. In the analysed area, the particles occurred individually and were surrounded by a continuous matrix, with no visible local porosity or discontinuities at the interfacial boundary. This does not imply the complete absence of agglomerates in the composite, as their local occurrence was demonstrated by SEM observations covering a substantially larger material area (Figure 2). The EDS maps additionally indicate a spatial correspondence between Mg- and O- enriched regions, particularly in the vicinity of the B_4_C particle. This may indicate Mg segregation in oxygen-containing regions or the presence of Mg-rich oxides. Hu et al. [30] reported the presence of MgO at and near B_4_C/Al interfaces in a B_4_C/Al-7093 composite, indicating that Mg binding in oxides may lead to local Mg depletion in the matrix and modify the precipitation process during aging. In contrast, Jiang et al. [31] confirmed the possibility of Mg segregation and the formation of Mg-, O-, and N-containing layers at B_4_C/Al interfaces. Local Cu-enriched regions may indicate the presence of phase constituents rich in this element, which is consistent with the complex phase composition of the Alumix 431 matrix and the identification of (Cu,Zn)Mg precipitates by HRTEM/FFT. The observed Mg- and O-enriched regions may therefore indicate local Mg binding in oxygen-containing interfacial regions, which could reduce the availability of Mg for precipitation of MgZn_2_ in the immediate vicinity of the B_4_C particles. This effect may become more relevant with increasing B_4_C content because of the larger total matrix–reinforcement interfacial area. Consequently, a possible reduction or modification of precipitation strengthening in interfacial regions may have partially contributed to the lower mechanical performance observed for the 10 wt.% B_4_C composite.

### 3.2. Hardness

**Figure 7:**
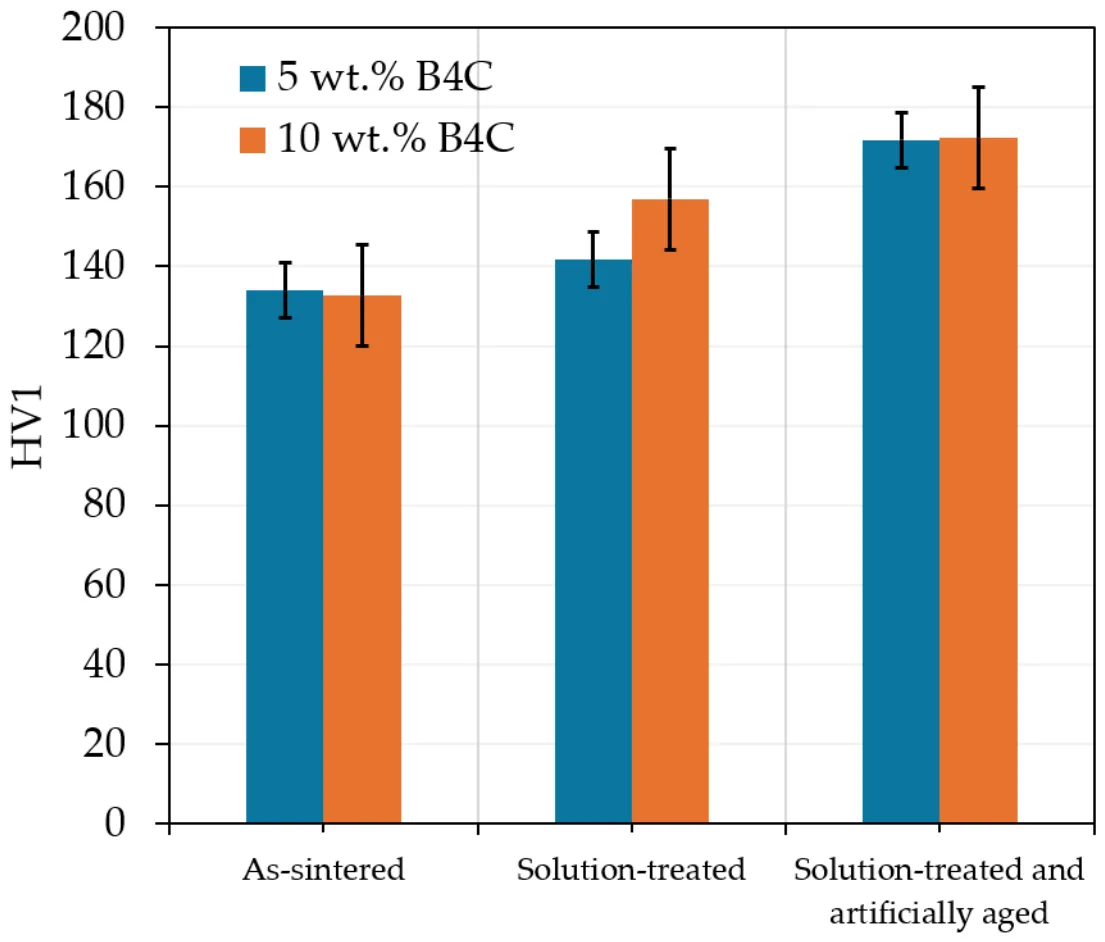
Vickers hardness (HV1) of the Alumix 431 composites containing 5 wt.% and 10 wt.% B_4_C in the as-sintered, solution-treated, and solution-treated and artificially aged conditions. Error bars represent standard deviations.

Figure 7 presents the hardness results of the Alumix 431–B_4_C composites in the investigated conditions. In both series, the highest hardness was obtained after solution treatment and artificial aging, reaching 172 HV1 for both the 5 and 10 wt.% B_4_C composites. Compared with the as-sintered FAST/SPS condition, this corresponds to an increase in hardness of approximately 28% and 29%, respectively. After solution treatment alone, the increase in hardness was more pronounced for the composite containing 10 wt.% B_4_C, which reached 157 HV1, than for the material with 5 wt.% B_4_C, for which the hardness was 142 HV1. However, the contribution of the individual mechanisms responsible for this change cannot be determined unambiguously from hardness measurements alone. It may result from the combined effects of solid-solution strengthening, undissolved secondary phases, ceramic particles, and the associated dislocations. Increasing the B_4_C content from 5 wt.% to 10 wt.% did not provide a systematic increase in hardness. In the as-sintered condition, the values for both composites were practically identical, and after aging they again reached the same level. This indicates that the final hardness was controlled primarily by the condition of the Alumix 431 matrix and the development of precipitation strengthening. At the same time, the composites containing 10 wt.% B_4_C showed a greater scatter in the results, consistent with the more heterogeneous particle distribution and the presence of local agglomerates observed in the microstructure.

**Figure 8:**
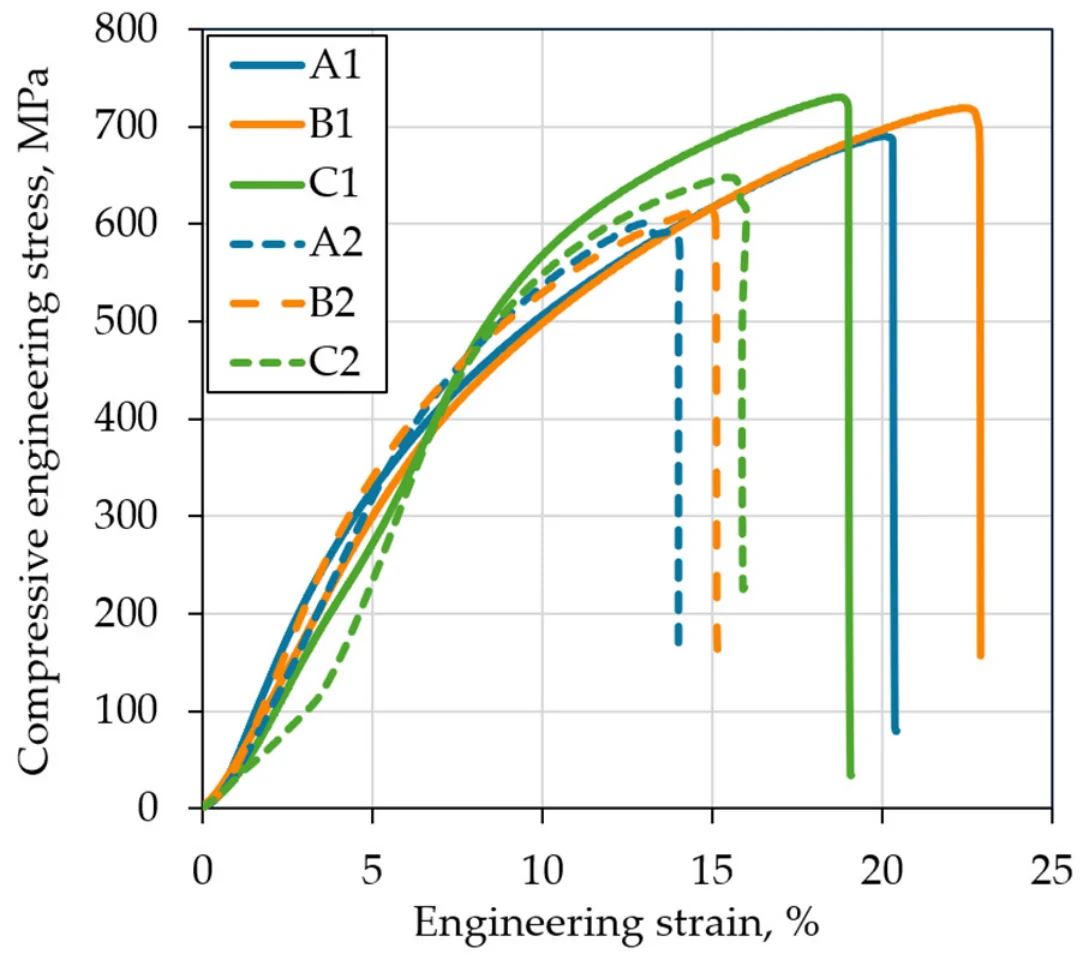
Representative engineering compressive stress–strain curves of the Alumix 431 composites containing 5 wt.% and 10 wt.% B_4_C in the as-sintered (A1 and A2), solution-treated (B1 and B2), and solution-treated and artificially aged (C1 and C2) conditions.

Representative engineering stress–strain curves are presented in Figure 8, while the stress values at 10% strain and the maximum recorded stress are summarised in Table 3. For the composite containing 5 wt.% B_4_C, heat treatment had a beneficial effect on the compressive properties. After solution treatment and artificial aging, the highest stress at 10% strain and maximum compressive stress were obtained, reaching 565 ± 21 MPa and 742 ± 12 MPa, respectively. Compared with the as-sintered FAST/SPS material, this represents a clear improvement in deformation resistance and compressive strength. A different trend was observed for the composites containing 10 wt.% B_4_C. Although their hardness after aging reached a level comparable to that of the materials containing 5 wt.% B_4_C, heat treatment did not result in an unambiguous improvement in compressive properties. The maximum recorded stress for the solution-treated and aged material was 564 ± 85 MPa, and the substantial standard deviation indicates greater variability in the mechanical response of this series. The shape of the stress–strain curves indicates that the investigated composites exhibited a limited ability to undergo stable plastic deformation. After reaching the maximum stress, a sharp decrease in stress occurred, characteristic of a brittle or quasi-brittle fracture mechanism. This effect was particularly evident in the composites containing 10 wt.% B_4_C, which fractured at lower strains and showed greater scatter in the results. This behaviour may be related to the greater heterogeneity in the distribution of the ceramic phase and the presence of local B_4_C particle agglomerates, which can act as local stress concentration sites and may facilitate crack initiation.

The combined analysis of the hardness and compression results indicates that, in the composite containing 5 wt.% B_4_C, solution treatment and artificial aging effectively increased the resistance of the matrix to deformation. The increase in hardness to 172 HV1 and in maximum compressive stress to 742 ± 12 MPa can be attributed primarily to the development of precipitation strengthening in the Alumix 431 matrix. TEM observations confirmed the presence of fine MgZn_2_ and (Cu,Zn)Mg precipitates, which hinder dislocation motion. A similar influence of the precipitation state on the properties of B_4_C/Al–Zn–Mg–Cu composites has been reported in the literature [22,23]. Increasing the B_4_C content to 10 wt.% did not result in a further improvement in the final hardness or compressive properties, although the hardness of both series reached 172 HV1 after aging. At the same time, the materials containing 10 wt.% B_4_C showed greater scatter in the results, limited deformability, and a higher tendency to fracture. This indicates that the potential strengthening effect of the higher ceramic-phase content was offset by its more heterogeneous distribution and by the presence of local B_4_C agglomerates, which promote stress concentration and crack initiation.

### 3.3. Wear and Friction Behaviour

The tribological behaviour of the Alumix 431–B_4_C composites was evaluated to determine the effects of B_4_C content and heat treatment on friction and wear under dry sliding conditions. The analysis included loss of mass, coefficient of friction, friction-force evolution, and examination of the worn surfaces, allowing the observed tribological response to be related to the microstructure and mechanical properties of the composites.

**Figure 9:**
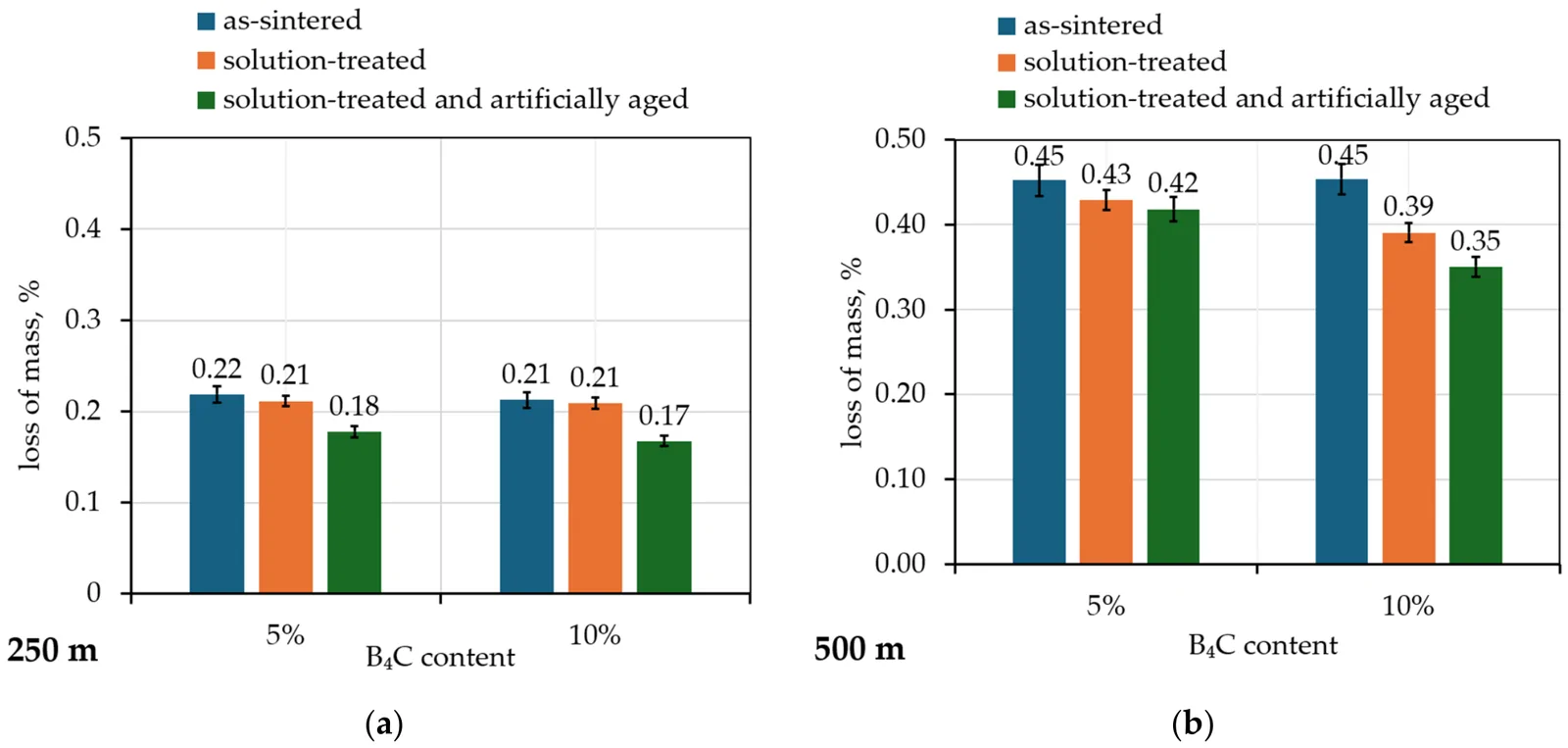
Effect of B_4_C content and heat treatment on the loss of mass of Alumix-based composites after dry sliding tests, (**a**) sliding distance of 250 m; (**b**) sliding distance of 500 m.

Figure 9 shows the loss of mass of the investigated composites after tribological testing as a function of B_4_C content and processing condition, i.e., in the as-sintered, solution-treated, and solution-treated and artificially aged states.

To eliminate the effect of different sliding distances on the wear results, the wear rate was calculated and expressed in g/N·m. Normalization of the material loss with respect to the applied normal load and the sliding distance allows direct comparison of the wear resistance between all investigated materials and testing conditions. Consequently, the wear rate provides a more representative measure of the intrinsic tribological performance than the loss of mass alone. The wear rate values calculated for all investigated material conditions are presented in Table 4.

The most notable finding of the present study is that increasing the B_4_C content from 5 to 10 wt.% did not result in a significant improvement in wear resistance in the as-sintered condition. The beneficial effect of the higher reinforcement content became evident only after heat treatment, particularly following artificial ageing and during the longer sliding distance of 500 m. These results indicate that the tribological performance of the investigated composites was governed not only by the amount of ceramic reinforcement but also by the interaction between the reinforcement particles and the aluminium matrix, whose mechanical response was substantially modified by precipitation strengthening.

At the shorter sliding distance of 250 m, only minor differences in loss of mass were observed between the composites reinforced with 5 and 10 wt.% B_4_C (Figure 9a). Artificial ageing reduced the loss of mass from 0.22 to 0.18 g for the composite containing 5 wt.% B_4_C, corresponding to a reduction of approximately 18.2%, whereas for the composite containing 10 wt.% B_4_C the loss of mass decreased from 0.21 to 0.17 g, representing a reduction of approximately 19.0%. After extending the sliding distance to 500 m, the influence of heat treatment became considerably more pronounced. For the composite containing 5 wt.% B_4_C, artificial ageing reduced the loss of mass from 0.45 to 0.42 g (6.7%), while for the composite reinforced with 10 wt.% B_4_C the reduction reached 22.2%, decreasing from 0.45 to 0.35 g. Consequently, the artificially aged composite reinforced with 10 wt.% B_4_C exhibited the lowest loss of mass among all investigated materials. The specific wear rate results (Table 4) followed the same overall tendency as the loss of mass measurements, confirming that the observed differences reflected a genuine improvement in wear resistance rather than only changes in the total amount of removed material. At the sliding distance of 250 m, artificial ageing reduced the wear rate by approximately 18.7% for the composite containing 5 wt.% B_4_C and by 21.3% for the composite reinforced with 10 wt.% B_4_C compared with the as-sintered condition. After extending the sliding distance to 500 m, the beneficial effect of heat treatment became considerably more pronounced for the composite containing 10 wt.% B_4_C. The wear rate decreased by approximately 22.8%, whereas the corresponding reduction for the composite containing 5 wt.% B_4_C reached only 7.6%. These results indicate that the synergistic effect of precipitation strengthening and ceramic reinforcement became increasingly effective with increasing sliding distance. The excellent agreement between the loss of mass and specific wear rate results confirms that the improved tribological performance originated from enhanced wear resistance rather than from differences associated with the testing procedure.

The obtained results differ from the trend commonly reported in the literature, where increasing the B_4_C content generally leads to improved wear resistance due to the high hardness of the ceramic particles and their ability to restrict deformation and removal of the aluminium matrix. Monikandan et al. [3] emphasized that the excellent tribological performance of Al–B_4_C composites results primarily from the high hardness of B_4_C particles, strong interfacial bonding and efficient load transfer between the matrix and the reinforcement. Similarly, Hynes et al. [6] demonstrated that increasing the B_4_C content from 5 to 15 wt.% in AA6061 composites resulted in higher hardness and improved wear resistance. Comparable improvements in wear resistance with increasing B_4_C content have also been reported by Şenel et al. [4] for sintered Al–B_4_C composites and by Raksha et al. [8] for Al2011–B_4_C composites, supporting the beneficial role of hard ceramic particles in restricting matrix deformation and material removal during sliding. In contrast, the present results indicate that increasing the reinforcement content alone was insufficient to improve wear resistance, since the composites containing 5 and 10 wt.% B_4_C exhibited almost identical loss of mass and specific wear rate in the as-sintered condition.

The microstructural observations revealed a relatively homogeneous distribution of B_4_C particles throughout the aluminium matrix for both reinforcement levels, although local particle agglomerates were occasionally observed in the composite containing 10 wt.% B_4_C. Therefore, the significant improvement in wear resistance after heat treatment cannot be explained solely by differences in particle distribution. Instead, the dominant factor appears to be the modification of the aluminium matrix during heat treatment. TEM/HRTEM observations confirmed the formation of fine MgZn_2_ and (Cu,Zn)Mg precipitates after artificial ageing, providing direct evidence of precipitation strengthening of the Al-Zn-Mg-Cu matrix. This interpretation is further supported by the increase in hardness and compressive strength observed after heat treatment. The precipitation-strengthened matrix exhibits greater resistance to local plastic deformation generated during sliding, allowing more effective support of the B_4_C particles. Consequently, a larger proportion of the external load can be transferred through the ceramic reinforcement (load-bearing effect), reducing the real contact area between the aluminium matrix and the steel counterface, in agreement with the mechanism proposed by Monikandan et al. [3].

Overall, the obtained results indicate that the effectiveness of the ceramic reinforcement depends not only on its content but also on the mechanical condition of the surrounding matrix. In the as-sintered condition, the relatively compliant aluminium matrix limited the reinforcing effect of the additional B_4_C particles, resulting in nearly identical wear behaviour for both composites. After artificial ageing, however, the precipitation-strengthened matrix was capable of efficiently supporting a larger number of ceramic particles, allowing the composite containing 10 wt.% B_4_C to fully exploit the advantages associated with the higher reinforcement fraction. This synergistic interaction explains why the greatest reductions in both loss of mass (22.2%) and specific wear rate (22.8%) were achieved only for the artificially aged composite containing 10 wt.% B_4_C after the 500 m sliding test. The surface phenomena responsible for these differences are further discussed based on the examination of the worn surfaces.

**Figure 10:**
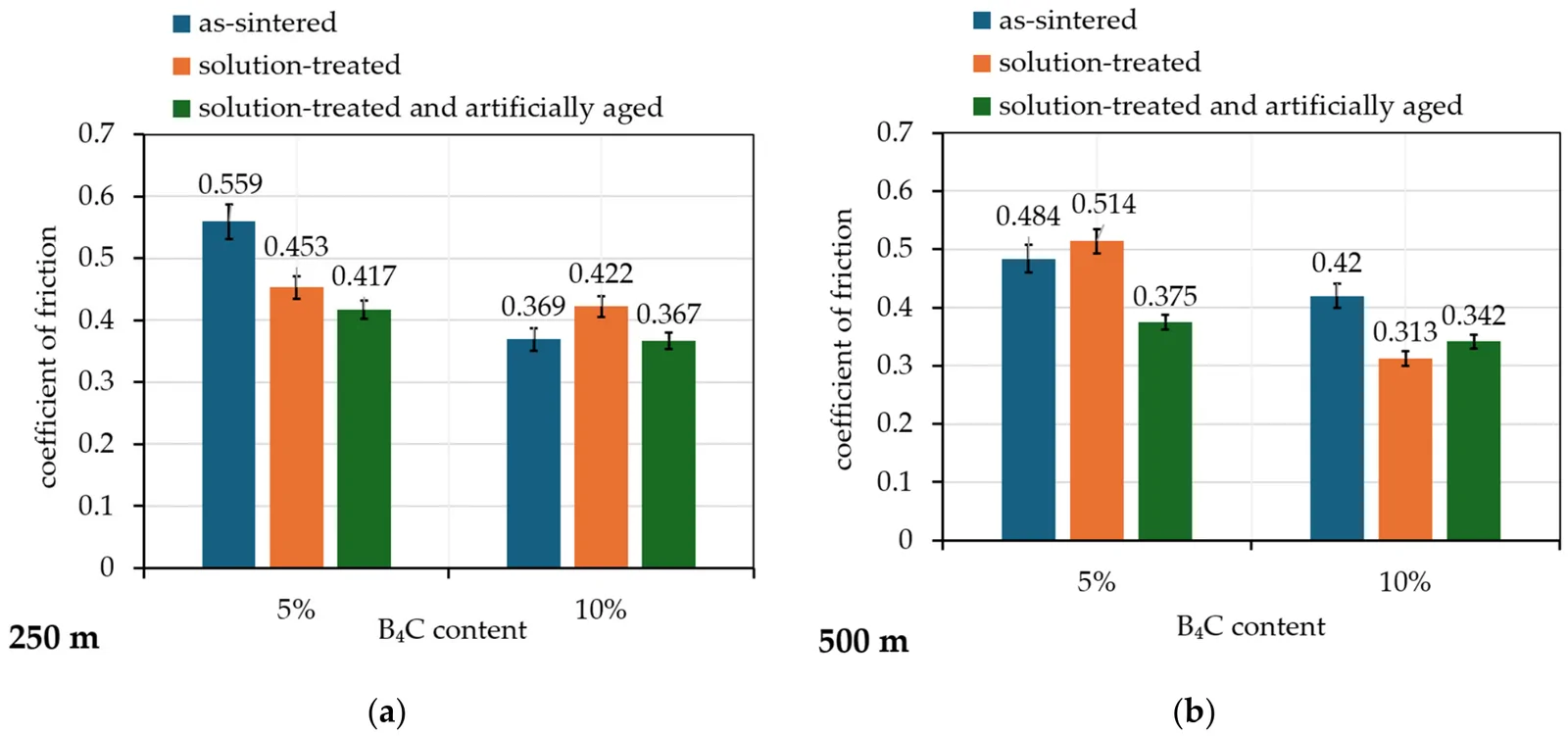
Effect of B_4_C content and heat treatment on the average coefficient of friction (COF) of Alumix-based composites: (**a**) sliding distance of 250 m; (**b**) sliding distance of 500 m.

The average coefficient of friction (COF) was used to assess the influence of B_4_C content and heat treatment on the frictional behaviour of the investigated composites. As an integral parameter describing the frictional response throughout the sliding test, the average COF enables direct comparison of the friction performance under different material conditions. The results obtained for sliding distances of 250 and 500 m are presented in Figure 10a and Figure 10b, respectively.

In contrast to the loss of mass and specific wear rate results, the COF values did not exhibit a simple monotonic relationship with either the B_4_C content or the applied heat treatment. Instead, they indicate that the frictional behaviour of the composites was governed by the evolution of the contact conditions during sliding rather than solely by the wear resistance of the material. After a sliding distance of 250 m, the composite containing 10 wt.% B_4_C exhibited consistently lower COF values than the material reinforced with 5 wt.% B_4_C. The average COF decreased from 0.559 to 0.369 in the as-sintered condition, corresponding to a reduction of approximately 34%. Following solution treatment, the difference became considerably smaller (0.453 and 0.422, respectively), whereas after artificial ageing both composites exhibited similarly low COF values of 0.417 and 0.367. These observations indicate that increasing the B_4_C content significantly modified the frictional response already during the shorter sliding distance, whereas the influence of heat treatment on the average COF remained comparatively limited. A different behaviour was observed after extending the sliding distance to 500 m. For the composite containing 5 wt.% B_4_C, solution treatment resulted in the highest average COF (0.514), exceeding both the as-sintered (0.484) and artificially aged (0.375) conditions. In contrast, the composite reinforced with 10 wt.% B_4_C exhibited the lowest COF after solution treatment (0.313), followed by artificial ageing (0.342) and the as-sintered condition (0.420). The reduction in COF between the as-sintered and solution-treated conditions reached approximately 25% for the composite containing 10 wt.% B_4_C, indicating that the frictional response became increasingly dependent on the interaction between the matrix and the reinforcing particles as the sliding distance increased.

It should be noted that the coefficient of friction did not directly correlate with the measured loss of mass and specific wear rate. For example, the composite containing 10 wt.% B_4_C in the as-sintered condition exhibited one of the lowest COF values despite showing wear resistance comparable to that of the composite reinforced with 5 wt.% B_4_C. Conversely, artificial ageing produced the lowest material loss even though the corresponding COF values were not always the minimum. This behaviour indicates that the coefficient of friction alone cannot be regarded as a direct measure of wear resistance, as friction and material removal are controlled by different mechanisms during sliding. In the present composites, the COF is strongly influenced by the evolving contact conditions, including the real contact area, surface deformation and the formation and removal of wear debris, whereas the specific wear rate is more directly associated with the extent of material removal. Therefore, changes in frictional resistance do not necessarily correspond to proportional changes in wear severity.

**Figure 11:**
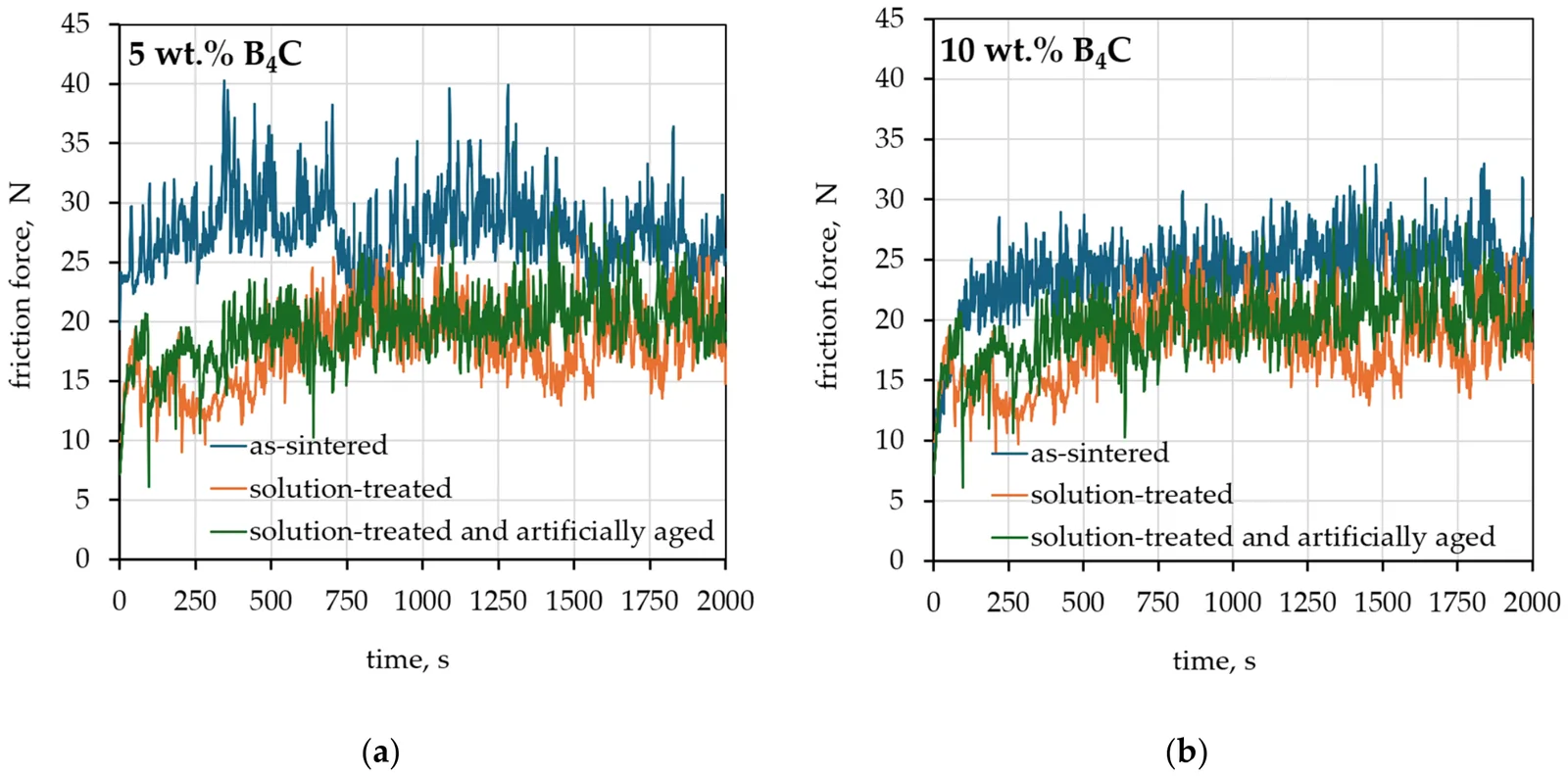
Friction force evolution during dry sliding tests over a sliding distance of 500 m for Alumix-based composites reinforced with: (**a**) 5 wt.% B_4_C; (**b**) 10 wt.% B_4_C.

To better characterise the friction behaviour, the friction-force evolution was recorded at both sliding distances. For clarity, only the results obtained after 500 m of sliding are presented (Figure 11). In the initial stage of sliding, the friction response is strongly influenced by the running-in process, during which the mating surfaces undergo mutual adaptation, asperity removal and stabilization of the real contact area. Consequently, the shorter test (250 m) contains a relatively larger contribution of the transient running-in stage, whereas the 500 m test provides a longer steady-state friction regime that better reflects the intrinsic tribological behaviour of the investigated composites. The extended sliding distance also enables evaluation of the stability of the tribological contact and the occurrence of fluctuations associated with wear debris formation, evolution of the tribological surface and changes in local contact conditions.

Regardless of the reinforcement content and heat-treatment condition, all materials exhibited a short running-in stage during the initial period of sliding, followed by the establishment of a quasi-steady friction regime. The running-in stage was associated with the progressive accommodation of the mating surfaces, removal of surface asperities, and gradual development of the real contact area. After this initial period, the friction force stabilized; however, both its average level and the magnitude of instantaneous fluctuations depended on the heat-treatment condition and the B_4_C content. For the composite containing 5 wt.% B_4_C (Figure 11a), the as-sintered material exhibited the highest friction force together with the largest fluctuation amplitude throughout the test. Numerous transient peaks observed after the running-in stage indicate repeated local disturbances of the sliding contact, suggesting cyclic changes in the real contact area during friction. Solution treatment significantly reduced the friction force and produced a more uniform signal. The artificially aged material exhibited intermediate friction force values; however, the fluctuations remained noticeably smaller than those observed for the as-sintered composite, indicating improved stability of the tribological contact. A similar tendency was observed for the composite reinforced with 10 wt.% B_4_C (Figure 11b). The as-sintered material again generated the highest friction force, whereas the solution-treated condition exhibited the lowest values over almost the entire sliding distance. Compared with the composite containing 5 wt.% B_4_C, the friction force signal was generally smoother, with fewer high-amplitude peaks, suggesting a more stable sliding process. Following artificial ageing, the friction force increased slightly compared with the solution-treated condition while maintaining relatively small fluctuations throughout the test.

Comparison of Figure 11a and Figure 11b indicates that increasing the reinforcement content from 5 to 10 wt.% B_4_C did not substantially alter the overall evolution of the friction force; however, it noticeably reduced the amplitude of instantaneous force fluctuations, particularly in the as-sintered condition. This behaviour suggests that the higher reinforcement content promoted more stable contact conditions during sliding. The influence of heat treatment was reflected primarily in the average friction-force level and signal stability. Solution treatment generally favoured lower friction forces, whereas artificial ageing resulted in slightly higher values but maintained relatively limited fluctuations. These differences demonstrate that the frictional response was governed not only by the mechanical properties of the matrix and the amount of B_4_C reinforcement, but also by the evolving conditions within the tribological contact. The contribution of matrix deformation, abrasive interaction and surface reactions occurring during sliding is discussed below on the basis of the worn-surface observations.

The friction-force results further demonstrate that friction and wear resistance are not directly correlated. Solution treatment generally favoured lower frictional resistance, whereas the greatest reduction in material loss was achieved after artificial ageing. This indicates that the material condition providing the lowest resistance to sliding is not necessarily the condition offering the highest resistance to material removal.

### 3.4. Analysis of Worn Surface Morphology and Wear Mechanisms

**Figure 12:**
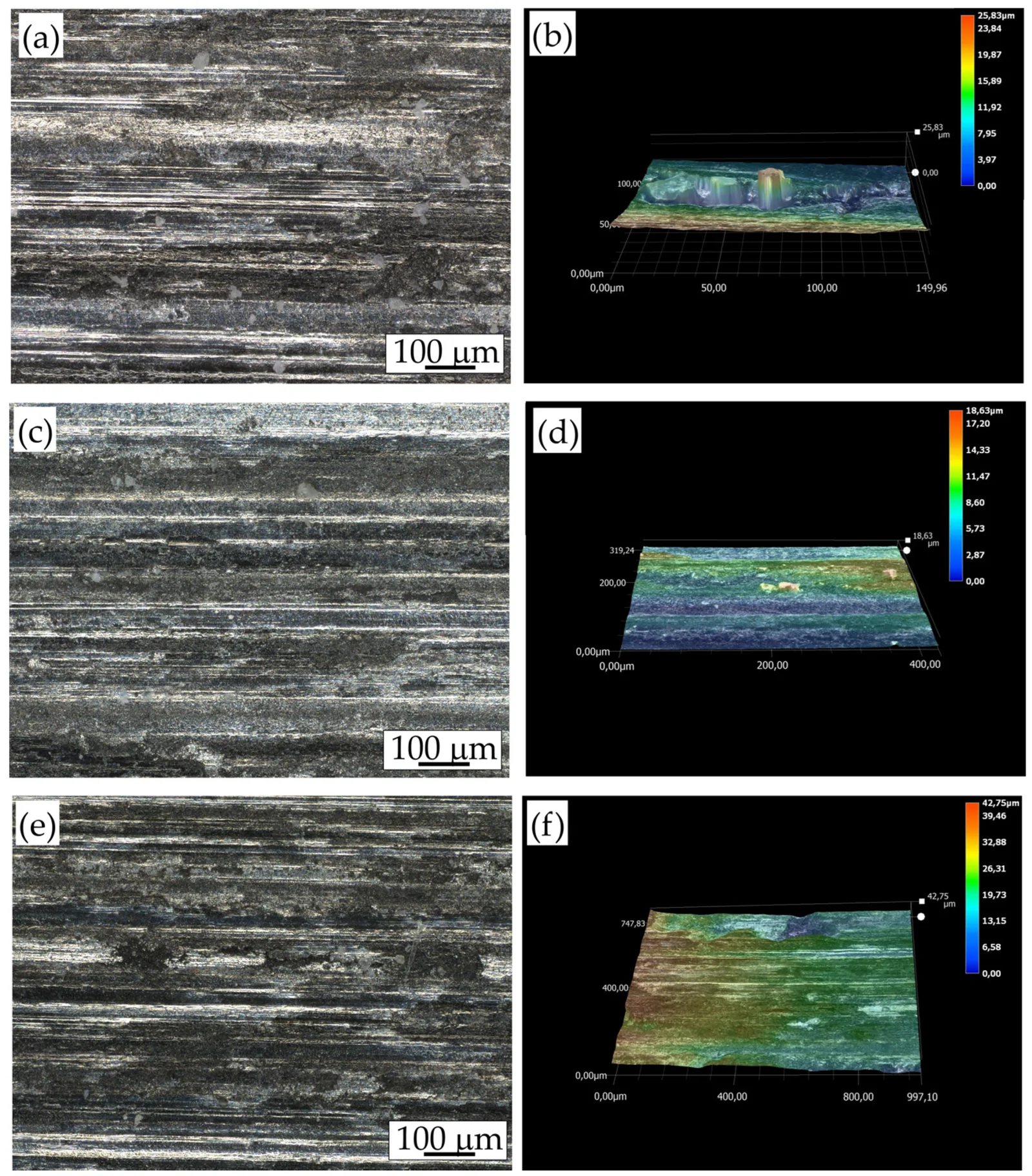
Worn surface morphologies and corresponding 3D surface topographies of the Alumix 431–5 wt.% B_4_C composite after a sliding distance of 500 m: (**a**,**b**) as-sintered, (**c**,**d**) solution-treated, and (**e**,**f**) solution-treated and artificially aged conditions.

The worn surfaces were examined using a digital microscope to assess their morphology and identify characteristic wear features. For the composite containing 5 wt.% B_4_C tested over a sliding distance of 500 m (Figure 12), the worn-surface morphology reveals that heat treatment affected primarily the severity and relative contribution of the wear mechanisms rather than causing a complete transition between fundamentally different wear modes. Grooves oriented parallel to the sliding direction are present in all three material conditions, indicating a persistent abrasive component. However, substantial differences are observed in the extent of plastic deformation, localization of surface damage and morphology of the regions formed during repeated sliding.

In the as-sintered condition (Figure 12a), the wear track is characterized by pronounced grooves accompanied by strongly deformed regions and displaced matrix material. The dominant mechanism can therefore be attributed to abrasive wear, primarily involving ploughing and a possible contribution of micro-cutting, accompanied by extensive plastic deformation of the aluminium matrix. The relatively compliant matrix facilitates penetration of the surface and displacement of material along the sliding direction. The heterogeneous character of the wear track is consistent with the relatively high loss of mass and specific wear rate determined for this condition and with the pronounced fluctuations in friction force observed during the test. These fluctuations may reflect repeated changes in the local contact conditions associated with deformation of the matrix, accumulation and removal of wear debris, and interaction of the counterface with locally exposed B_4_C particles. Considering the aluminium-rich matrix and dry-sliding conditions, local oxidation of freshly exposed and mechanically activated surface regions is also likely to accompany this process, although its contribution cannot be directly quantified from the morphological observations alone. After solution treatment (Figure 12b), longitudinal grooves remain clearly visible, confirming that abrasion continues to control material removal. However, the wear track is more homogeneous and plastic deformation appears to be distributed more uniformly over the contact area. The wear mechanism can therefore be described as abrasive wear accompanied by more homogeneous plastic deformation of the matrix and a probable tribo-oxidative contribution. Compared with the as-sintered condition, the reduced localization of severe surface deformation is consistent with the relatively small decrease in loss of mass and specific wear rate. At the same time, the coefficient of friction does not decrease proportionally, demonstrating that the modest improvement in wear resistance cannot be explained simply by reduced frictional resistance. Instead, solution treatment primarily modifies the manner in which the matrix accommodates contact stresses, while its susceptibility to abrasive removal remains significant. Following artificial ageing (Figure 12c), abrasive grooves remain present, showing that the abrasive component of wear is not eliminated. However, extensive plastic flow of the matrix is less pronounced and the damage becomes more localized. A similar effect of B_4_C reinforcement has been reported for aluminium matrix composites, where hard ceramic particles restricted plastic flow of the matrix and reduced the severity of grooving during sliding [8]. The prevailing mechanism can therefore be interpreted as less severe abrasive wear accompanied by reduced matrix plastic deformation and probable tribo-oxidative processes. This change is consistent with the lowest loss of mass and specific wear rate measured for the 5 wt.% B_4_C composite and with the favourable friction behaviour observed after ageing. Thus, artificial ageing does not fundamentally replace abrasion with another wear mechanism but reduces its severity and modifies the conditions under which the tribological contact develops.

The improved tribological response after artificial aging can therefore be attributed to the combined effect of precipitation strengthening of the Alumix 431 matrix and B_4_C reinforcement. The strengthened matrix limits local plastic deformation during sliding and provides more effective mechanical support for the B_4_C particles. This enhances their contribution to load bearing and restricts penetration and ploughing of the aluminium-rich matrix. Thus, artificial aging improves wear resistance without fundamentally changing the dominant abrasive wear mechanism.

**Figure 13:**
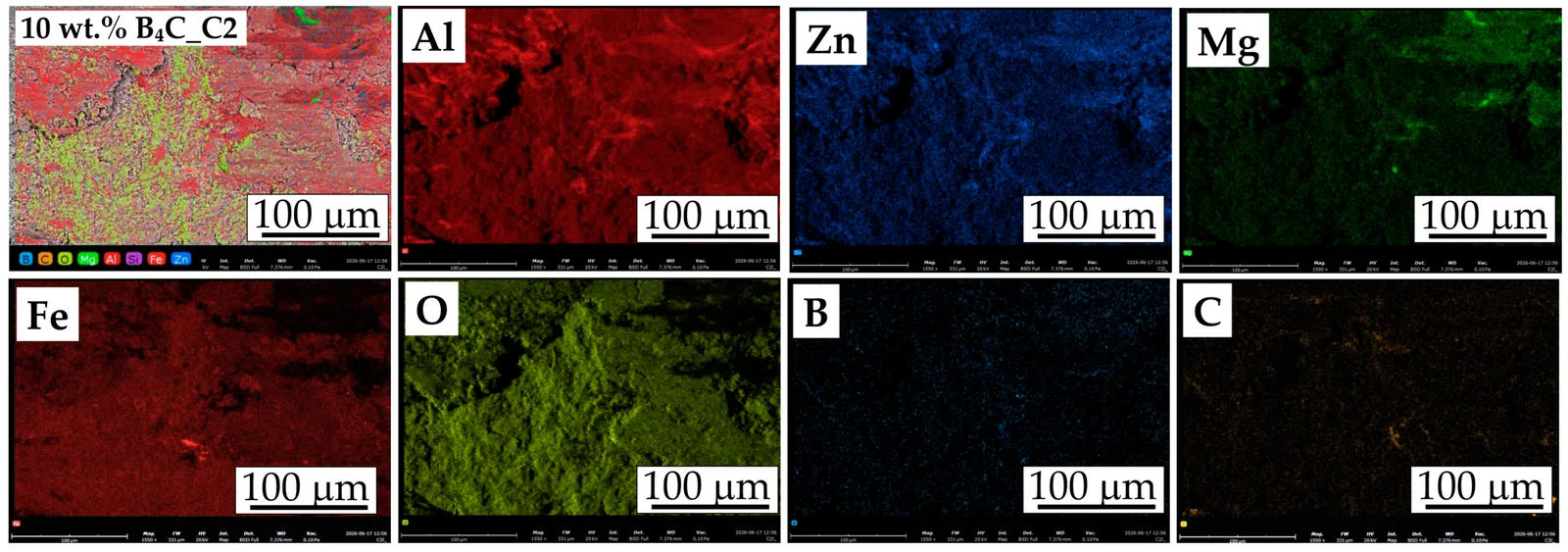
SEM image and corresponding EDS elemental maps of the worn surface of the Alumix 431–10 wt.% B_4_C composite after solution treatment and artificial aging and a sliding distance of 500 m.

To assess the role of surface oxidation and possible material transfer in the wear process, EDS elemental distribution maps were acquired from the worn surface of the artificially aged 10 wt.% B_4_C composite after 500 m of sliding (Figure 13).

A pronounced oxygen signal is distributed over extensive regions of the wear track, directly confirming that tribo-oxidation accompanies the mechanical wear processes under the applied dry-sliding conditions. The occurrence of oxidation during dry sliding is consistent with previous observations for aluminium-based B_4_C composites, where oxide-containing regions were identified on worn surfaces [8]. The oxygen distribution is spatially heterogeneous rather than uniform, suggesting repeated formation, disruption and redistribution of oxidized material during sliding rather than the development of a continuous and fully protective oxide film. Such behaviour is consistent with a dynamic tribological surface in which abrasion continuously exposes fresh matrix material, while frictional interaction promotes its subsequent oxidation. Importantly, Fe is detected only in isolated regions of the wear track, indicating that transfer of material from the steel counterface is limited and does not result in the formation of a continuous Fe-rich transfer layer. Therefore, the improved tribological response of the heat-treated composite should not be attributed primarily to counterface-material transfer. Instead, the EDS results support a combined mechanism involving abrasive wear, tribo-oxidation and restricted plastic deformation of the precipitation-strengthened matrix. The oxidized regions may additionally reduce the extent of direct metallic contact between the aluminium-rich matrix and the steel counterface, thereby contributing to the favourable friction behaviour, although the present EDS data do not demonstrate the formation of a continuous protective tribolayer.

Overall, the combined morphological, tribological and EDS results indicate that abrasion and tribo-oxidation coexist during dry sliding, while heat treatment primarily controls the severity of matrix deformation and material removal. In the as-sintered condition, pronounced ploughing and extensive plastic deformation produce a relatively severe and unstable wear process. Solution treatment results in a more homogeneous surface response but only moderately limits abrasive removal. Artificial ageing produces the most favourable combination: precipitation strengthening restricts extensive matrix deformation and improves the mechanical support of B_4_C particles, while localized tribo-oxidative processes accompany the remaining abrasive interaction. The resulting evolution of the wear process is fully consistent with the reduction in loss of mass and specific wear rate and with the corresponding changes in friction behaviour. The observed response is therefore consistent with the expected behaviour of a precipitation-strengthened, ceramic-particle-reinforced aluminum matrix composite, in which improved matrix support increases the effectiveness of the hard reinforcement in resisting material removal.

## 4. Conclusions

Based on the results obtained, the following conclusions can be drawn:

1. This study demonstrates that the performance of Alumix 431–B_4_C composites is governed not only by the B_4_C content but also by the microstructural condition of the precipitation-hardenable matrix induced by heat treatment. The applied experimental design made it possible to distinguish the effects of reinforcement content and solution treatment followed by artificial aging on the microstructure and mechanical response of the composites.
2. The B_4_C reinforcement was located predominantly in regions corresponding to the boundaries of the original Alumix 431 powder particles. Increasing the B_4_C content to 10 wt.% did not provide a further improvement in the final hardness or compressive properties, which may be related to the increased microstructural heterogeneity and the presence of local B_4_C agglomerates that can act as stress concentration sites.
3. Artificial aging produced fine MgZn_2_ and (Cu,Zn)Mg precipitates in the Alumix 431 matrix, as confirmed by TEM/HRTEM and electron diffraction. This precipitation response was associated with the substantial increase in hardness and compressive strength of the composite containing 5 wt.% B_4_C.
4. The most favourable mechanical response was obtained for the 5 wt.% B_4_C composite after solution treatment and artificial aging, reaching 172 HV1 and a maximum compressive stress of 742 ± 12 MPa. Increasing the B_4_C content to 10 wt.% did not provide a further improvement in the final hardness or compressive properties, because the potential strengthening effect was offset by microstructural heterogeneity and local stress concentrators.
5. Increasing the B_4_C content from 5 wt.% to 10 wt.% did not significantly improve the wear resistance in the as-sintered condition. The beneficial effect of the higher reinforcement content became evident after heat treatment, particularly following artificial aging and at the longer sliding distance of 500 m. Under these conditions, the 10 wt.% B_4_C composite exhibited the lowest loss of mass and specific wear rate, demonstrating a synergistic effect between precipitation strengthening of the matrix and the higher ceramic reinforcement content.
6. The coefficient of friction did not directly correlate with loss of mass or specific wear rate, demonstrating that frictional resistance and material removal were governed by different aspects of the evolving tribological contact. Increasing the B_4_C content generally promoted a more stable friction response, while heat treatment modified both the average friction level and the amplitude of friction-force fluctuations. Thus, the lowest coefficient of friction did not necessarily correspond to the condition exhibiting the highest wear resistance.
7. Worn-surface observations revealed that abrasive wear, primarily associated with ploughing and plastic deformation of the aluminium matrix, remained an important wear mechanism in all investigated conditions. Artificial aging reduced the severity of matrix deformation and material removal by strengthening the matrix and improving the mechanical support of the B_4_C particles. EDS mapping additionally confirmed the contribution of tribo-oxidation during sliding, indicating that the improved tribological performance resulted from the combined effects of matrix strengthening, ceramic reinforcement, and evolution of the tribological surface.

## Figures and Tables

**Table 1:** Chemical composition of Alumix 431 powder (wt.%).

Element	Zn	Mg	Cu	Sn	Al
Content, wt.%	6.4	2.80	1.80	0.29	Balance

**Table 2:** Sample designations and processing and heat-treatment conditions.

Sample	B_4_C Content, wt.%	Material Condition	Processing/Heat-Treatment Conditions
A1	5	As-sintered	FAST/SPS: 500 °C, 10 min
B1	5	Solution-treated	480 °C, 1 h; water quenching
C1	5	Solution-treated and artificially aged	480 °C, 1 h; water quenching; 160 °C, 8 h
A2	10	As-sintered	FAST/SPS: 500 °C, 10 min
B2	10	Solution-treated	480 °C, 1 h; water quenching
C2	10	Solution-treated and artificially aged	480 °C, 1 h; water quenching; 160 °C, 8 h

**Table 3:** Hardness and compressive properties of Alumix 431–B_4_C composites as a function of B_4_C content and material condition.

Sample	Stress at 10% Engineering Strain, MPa	Maximum Recorded Stress, MPa
A1	470 ± 37.5	688 ± 3
B1	507 ± 5	707 ± 6
C1	565 ± 1	742 ± 12
A2	537 ± 54	597 ± 68
B2	497 ± 33	604 ± 10
C2	501 ± 49	564 ± 85

**Table 4:** Specific wear rate of Alumix 431–B_4_C composites as a function of B_4_C content and material condition.

B_4_C Content wt.%	Sliding Distance	As-Sintered	Solution-Treated	Artificially Aged
5%	250 m	1.535 × 10^−5^	1.483 × 10^−5^	1.248 × 10^−5^
	500 m	1.587 × 10^−5^	1.505 × 10^−5^	1.467 × 10^−5^
10%	250 m	1.491 × 10^−5^	1.467 × 10^−5^	1.174 × 10^−5^
	500 m	1.592 × 10^−5^	1.371 × 10^−5^	1.229 × 10^−5^

## Data Availability

The original contributions presented in this study are included in the article. Further inquiries can be directed to the corresponding authors.

## Abbreviations

The following abbreviations are used in this manuscript:

FAST/SPS	Field-assisted sintering technology/spark plasma sintering
SEM	Scanning electron transmission microscopy
TEM	Transmission electron microscopy
HRTEM	High resolution transmission microscopy
COF	Coefficient of friction
EDS	Energy-dispersive X-ray spectroscopy
XRD	X-ray diffraction
HV1	Vickers hardness measured using a test force of 1 kgf (9.807 N)

## References

[1] Jung J., Kang S. (2004). Advances in Manufacturing Boron Carbide-Aluminum Composites. J. Am. Ceram. Soc..

[2] Lloyd D.J. (1994). Particle reinforced aluminium and magnesium matrix composites. Int. Mater. Rev..

[3] Monikandan V.V., Pratheesh K., Rajendrakumar P.K., Joseph M.A. (2022). Towards the Enhanced Mechanical and Tribological Properties and Microstructural Characteristics of Boron Carbide Particles Reinforced Aluminium Composites: A Short Overview. Johns. Matthey Technol. Rev..

[4] Şenel M.C., Kanca Y., Gürbüz M. (2022). Reciprocating sliding wear properties of sintered Al–B_4_C composites. Int. J. Miner. Met. Mater..

[5] Shirvanimoghaddam K., Khayyam H., Abdizadeh H., Akbari M.K., Pakseresht A., Ghasali E., Naebe M. (2016). Boron carbide reinforced aluminium matrix composite: Physical, mechanical characterization and mathematical modelling. Mater. Sci. Eng. A.

[6] Hynes N.R.J., Raja S., Tharmaraj R., Pruncu C.I., Dispinar D. (2020). Mechanical and tribological characteristics of boron carbide reinforcement of AA6061 matrix composite. J. Braz. Soc. Mech. Sci. Eng..

[7] Beder M. (2025). The effect of high B_4_C ratio on the improvement of mechanical properties and wear resistance of Al2024/B_4_C composites fabricated by mechanical milling-assisted hot pressing. Ceram. Int..

[8] Raksha M.S., Adaveesh B., Nagaral M., Boppana S.B., Anjinappa C., Khan M.S., Wahab M.O.A., Islam S., Bhardwaj V., Palavalasa R.K. (2023). Impact of boron carbide particles and weight percentage on the mechanical and wear characterization of Al2011 alloy metal composites. ACS Omega.

[9] Sha G., Cerezo A. (2004). Early-stage precipitation in Al–Zn–Mg–Cu alloy (7050). Acta Mater..

[10] Ma K., Hu T., Yang H., Topping T., Yousefiani A., Lavernia E.J., Schoenung J.M. (2016). Coupling of dislocations and precipitates: Impact on the mechanical behavior of ultrafine grained Al–Zn–Mg alloys. Acta Mater..

[11] Ma K., Wen H., Hu T., Topping T.D., Isheim D., Seidman D.N., Lavernia E.J., Schoenung J.M. (2014). Mechanical behavior and strengthening mechanisms in ultrafine grain precipitation-strengthened aluminum alloy. Acta Mater..

[12] Massardier V., Merle P. (1998). Mechanisms of interaction controlling the kinetics of zone formation in metal matrix composites: Comparison of the effect of the reinforcement in Al–Cu and Al–Mg–Si matrix composites. Mater. Sci. Eng. A.

[13] Massardier V., Pelletier L., Merle P. (1998). Influence of the introduction of ceramic particles in Al–Cu alloys on GP zone formation. Mater. Sci. Eng. A.

[14] Xue W., Jiang L., Zhang B., Jing D., He T., Chen G., Xiu Z., Wu G. (2020). Quantitative analysis of the effects of particle content and aging temperature on aging behavior in B_4_C/6061Al composites. Mater. Charact..

[15] Li Y.Z., Wang Q.Z., Wang W.G., Xiao B.L., Ma Z.Y. (2015). Effect of interfacial reaction on age-hardening ability of B_4_C/6061Al composites. Mater. Sci. Eng. A.

[16] Wu C., Ma K., Zhang D., Wu J., Xiong S., Luo G., Zhang J., Chen F., Shen Q., Zhang L. (2017). Precipitation phenomena in Al–Zn–Mg alloy matrix composites reinforced with B_4_C particles. Sci. Rep..

[17] Chen C., Li B., Wang Y., Bian M., Kang X., Yang X. (2025). Recent advances on aluminum-based boron carbide composites: Performance, fabrication, and applications. Materials.

[18] Arslan G., Kara F., Turan S. (2003). Quantitative X-ray diffraction analysis of reactive infiltrated boron carbide–aluminium composites. J. Eur. Ceram. Soc..

[19] Toptan F., Kilicarslan A., Karaaslan A., Cigdem M., Kerti I. (2010). Processing and microstructural characterisation of AA 1070 and AA 6063 matrix B_4_C_p_ reinforced composites. Mater. Des..

[20] Sohail G., Kusar A., Aziz T., Mohsin A., Fatima N., Shahid R.N., Tariq N.H., Zarif M., Asghar Z., Ali F. (2026). Tailoring properties of high-performance B_4_C/6061Al metal matrix composites through efficient heat treatment. J. Compos. Mater..

[21] Wąsik A., Leszczyńska-Madej B., Madej M., Rubach R., Garbiec D. (2022). Effect of holding time on densification, microstructure and selected properties of spark plasma sintered AA7075–B_4_C composites. Materials.

[22] Chen Y.T., Zhu S.Z., Luo H.J., Wang D., Xiao B.L., Ma Z.Y. (2024). Effects of solution temperatures on microstructures and mechanical properties of B_4_C/7A04Al composites: A comparison study with 7A04Al alloys. Mater. Sci. Eng. A.

[23] Chen Y.T., Ma G.N., Zhu S.Z., Wang D., Xiao B.L., Ma Z.Y. (2024). Two-stage aging treatment to accelerate aging kinetics without impairing strength in B_4_C/7A04Al composite. Sci. China Technol. Sci..

[24] Leszczyńska-Madej B., Madej M., Wąsik A. (2023). Analysis of sintering process of Alumix431–B_4_C composites. J. Alloys Compd..

[25] (2018). Metallic Materials — Vickers Hardness Test — Part 1: Test Method.

[26] (2024). Standard Test Methods of Compression Testing of Metallic Materials at Room Temperature.

[27] Xu P., Feng Y., Xu B., Chen F., Lin Y. (2017). Disruption of oxide films during spark plasma sintering of micrometric-sized Al powders and its effect on microstructure and tensile properties of the consolidated Al. J. Alloys Compd..

[28] Leszczyńska-Madej B., Madej M., Wąsik A. (2026). Microstructural pathways to interface stability in Al–SiC metal matrix composites: Powder metallurgy versus field-assisted sintering/spark plasma sintering consolidation. Adv. Eng. Mater..

[29] Gruber R., Singewald T.D., Bruckner T.M., Hader-Kregl L., Hafner M., Groiss H., Duchoslav J., Stifter D. (2023). Investigation of oxide thickness on technical aluminium alloys—A comparison of characterization methods. Metals.

[30] Hu H.M., Lavernia E.J., Harrigan W.C., Kajuch J., Nutt S.R. (2001). Microstructural investigation on B_4_C/Al-7093 composite. Mater. Sci. Eng. A.

[31] Jiang L., Wen H., Yang H., Hu T., Topping T.D., Zhang D.L., Lavernia E.J., Schoenung J.M. (2015). Influence of length-scales on spatial distribution and interfacial characteristics of B_4_C in a nanostructured Al matrix. Acta Mater..

